# Landscape of gene fusions in hormone receptor-positive breast cancer reveals *ADK* fusions as drivers of progression and potential therapeutic targets

**DOI:** 10.1038/s41421-025-00830-z

**Published:** 2025-11-11

**Authors:** Yang Ou-Yang, Ding Ma, Cai-Jin Lin, Yun-Song Yang, Cheng-Lin Liu, Jing Hou, Xi Jin, Zhi-Ming Shao, Yi-Zhou Jiang

**Affiliations:** 1 Key Laboratory of Breast Cancer in Shanghai, Department of Breast Surgery, Fudan University Shanghai Cancer Center, Shanghai, China; 2 Department of Breast Surgery, Guizhou Provincial People’s Hospital, Guiyang, Guizhou China; 3 Shanghai Academy of Natural Sciences (SANS), Fudan University, Shanghai, China

**Keywords:** Breast cancer, Growth factor signalling, Comparative genomics

## Abstract

Gene fusions are becoming critical oncogenic drivers with potential therapeutic relevance across various cancers. However, their roles and clinical implications in breast cancer remain largely unexplored. In this study, we leveraged a large-scale multiomics cohort and a drug screening platform for breast cancer to systematically profile gene fusions. We identified *ADK* fusion genes as novel and recurrent drivers in hormone receptor-positive (HR+)/human epidermal growth factor receptor 2-negative (HER2‒) breast cancer. Functionally, the most commonly occurring *ADK* fusion gene, *KAT6B::ADK*, enhances metastatic potential and confers tamoxifen resistance. Mechanistically, *KAT6B::ADK* activates ADK kinase activity through liquid‒liquid phase separation, triggering the activation of an integrated stress response signaling pathway. Notably, patient-derived organoids harboring *KAT6B::ADK* from HR+/HER2‒ breast cancer demonstrate increased sensitivity to ADK inhibitors, underscoring the therapeutic potential of this fusion gene. Our findings establish *ADK* fusions as therapeutic targets in HR+/HER2‒ breast cancer, offering new avenues for innovative precision treatment strategies in this patient population.

## Introduction

Gene fusions, arising from genomic translocations, insertions, deletions, or chromosomal inversions, constitute a class of molecular aberrations with significant implications in cancer biology. A substantial proportion of gene fusions drive tumorigenesis and/or promote tumor progression. More importantly, gene fusions represent strong potential targets for targeted therapies. The advent of targeted therapies against constitutively activated oncogenic kinases, such as those resulting from gene fusions, has shown remarkable efficacy in various cancer types^1^. The discovery of novel gene fusions in epithelial tumors has had significant therapeutic effects in recent years. This is represented by the discovery of an *EML4::ALK* fusion in ~4% of lung cancers and an *FGFR::TACC* fusion in ~3% of glioblastomas, which have culminated in effective targeted therapies for these tumors^2,3^. Most recently, Larotrectinib, which targets the *NTRK* gene fusions and accounts for up to ~1% of solid tumors, has received approval from the Food and Drug Administration for pancancer use and is considered the first targeted therapy with tissue-agnostic indications^4^. Although low in percentage, these neoplastic gene fusions can be applied to genetic subtyping of solid tumors that may be curable by fusion-targeted therapies. Therefore, gene fusion identification is valuable not only for understanding the biological mechanisms of tumorigenesis but also for providing significant clinical opportunities to treat cancers.

In the context of breast cancer, gene fusions are common^5–7^. However, the identification of fusion genes with significant pathological implications remains strikingly rare. This phenomenon can be attributed to several factors. First, the technical challenges involved in distinguishing genuine fusion events from false positives have historically hindered the discovery of large-scale gene fusions^8,9^. Advancements in next-generation sequencing technologies and analytical pipelines have gradually addressed these limitations, making the identification of fusions in breast cancer more feasible. Second, the low frequency of individual fusion events dampens researchers’ interest in exploring the functional roles of these genes. Consequently, most studies have focused on identifying fusion genes through DNA or RNA sequencing, with limited emphasis on validating their oncogenic potential or role in modulating therapeutic responses^10–12^. Additionally, most research is constrained by small sample sizes and a focus on a single omics dimension, which restricts our comprehensive understanding of the landscape of fusion genes in breast cancer and their impact on disease progression^13–16^. Therefore, well-annotated multiomics cohorts combined with the functional characterization of fusion genes are essential for identifying fusion events with genuine clinicopathological significance.

In this study, we utilized large-scale multiomics cohorts (FUSCC-BRCA and FUSCC-PDOs [patient-derived organoids]) to comprehensively explore the biological characteristics and clinical relevance of fusion genes. By integrating genomic, transcriptomic, and metabolomic data, we identified novel fusion genes with biological and clinical significance in breast cancer. Our findings provide a valuable resource for functional and translational research on fusion genes in breast cancer and highlight their potential as predictive biomarkers and therapeutic targets.

## Results

### Landscape of fusion genes in breast cancer

To systematically explore the biological and clinical relevance of fusion genes in breast cancer, we established a large-scale multiomics cohort (FUSCC-BRCA) and a cohort of PDOs for drug sensitivity testing (FUSCC-PDOs).

**Fig. 1:**
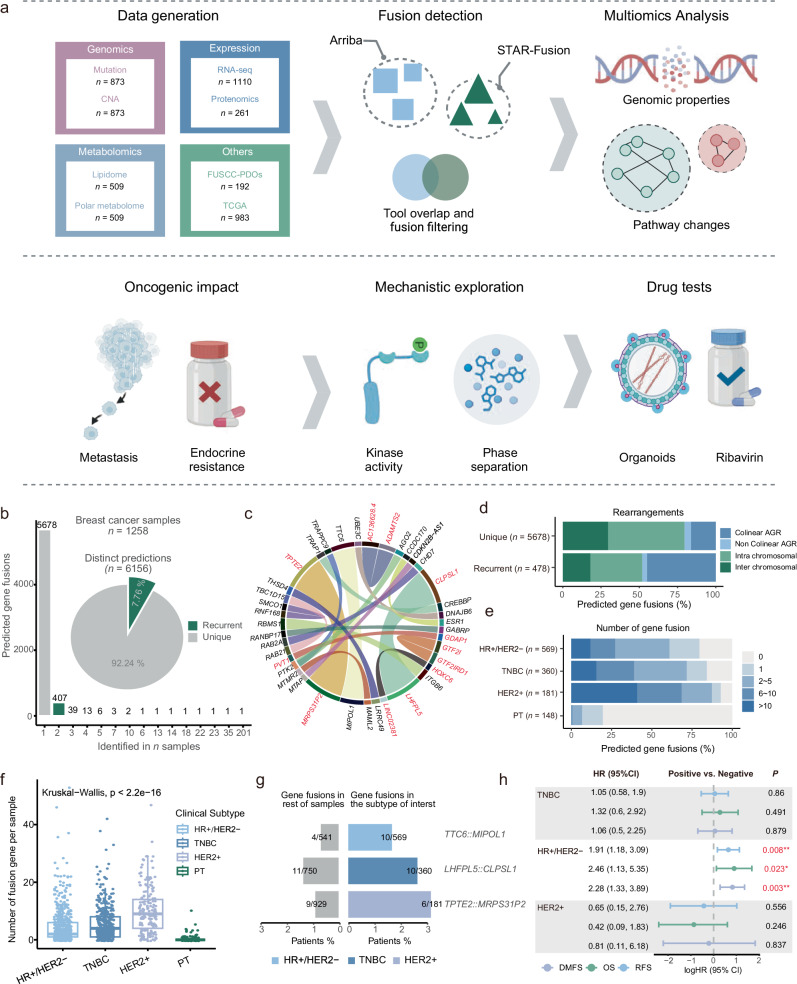
Schematic overview of the study design and the landscape in HR+/HER2‒ breast cancer. **a** Schematic overview of the study design. **b** The frequency of recurrence is shown for distinct gene fusions in 1361 breast cancer samples (bar chart). The proportions of recurrently (green) and uniquely (gray) predicted gene fusions are also depicted (pie chart). **c** Circles represent the landscape of the fusion genes. Recurrent fusions (more than four samples) are displayed as connected gene pairs, in which the width of the connecting arc represents the number of samples containing the fusion. Red highlights novel gene fusions that are absent from public databases (FusionGDB 2.0 and ChimerDB 4.0). **d** The number of recurrent and unique gene fusions is shown according to the configuration type of the breakpoints. “Adjacent gene rearrangements (AGR)” indicates genomic rearrangements between adjacent genes (genes within a 500-kb distance). “Interchromosomal” indicates genomic rearrangements on different chromosomes. “Noncolinear” indicates breakpoints on different strands. “Colinear AGRs” indicates gene fusions with breakpoints on the same chromosome and strand within a 500-kb distance, whereas “intrachromosomal” indicates gene fusions with breakpoints farther apart. The percentages and total number of gene fusions are indicated. **e** The data show the proportions of breast cancer immune histochemistry (IHC) subtypes with > 10, 6–10, 2–5, 1, and 0 fusion genes. The number of samples analyzed for each tumor type is given in parentheses. **f** Distribution of the number of fusion genes in different breast cancer IHC subtypes. **g** Comparison of the prevalence of gene fusions in different breast cancer IHC subtypes. The number of patients with each fusion and the total number of patients with each breast cancer IHC subtype are shown on each bar. **h** Prognostic significance of fusion genes in different breast cancer IHC subtypes.

The FUSCC-BRCA cohort included the multiomics data, clinicopathological details, and clinical outcomes of 1226 breast cancer patients. A total of 873 patients had whole-exome sequencing data and somatic copy number alteration (CNA) data from primary tumor tissues and paired blood samples. RNA sequencing data were available for 1110 patients, tandem mass tag-based quantitative proteomics data for 261 patients, and metabolomics data for 509 patients (Fig. 1a). This cohort was used to delineate the landscape of fusion genes in breast cancer and to characterize their molecular biological features on the basis of detailed annotations and comprehensive multiomics data. To comprehensively depict the landscape of gene fusions in breast cancer, we further incorporated the TCGA-BRCA cohort (*n* = 983) for external validation and biological characterization. Additionally, we investigated the biological functions of gene fusions in breast cancer progression both in vitro and in vivo. Leveraging the FUSCC-PDOs cohort (*n* = 192), we employed RT-PCR to identify fusion genes, conducted subsequent drug sensitivity analyses, and proposed targeted therapeutic strategies for clinical use (Fig. 1a).

Through the application of a bioinformatics pipeline to analyze fusion genes in RNA sequencing data, we identified a total of 6156 fusion genes. The majority of these were unique fusions, appearing only once across all the samples (5678/6156, 92.24%). Recurrent fusions, which occurred two or more times, represented a relatively small subset (478/6156, 7.76%) (Fig. 1b). Our database identified well-known fusion genes that are commonly associated with breast cancer, such as *ESR1::CCDC170*, and revealed previously unreported fusion genes, including *TPTE2::MRPS31P2* and *LHFPL5::CLPSL1* (Fig. 1c). We observed that ~half (44.98%) of the breakpoints were located in a *cis*-near configuration (same chromosome, same strand, and within 500 kilobases (kb)) (Fig. 1d). We further investigated the presence of fusion genes in the breast cancer samples and reported that the majority of the breast cancer tissues harbored fusion genes (925/1110, 83.33%), whereas only a small proportion of the paratumor tissues harbored fusion genes (29/148, 19.6%) (Fig. 1e). Among breast cancer subtypes, the highest frequency of fusion genes was observed in the hormone receptor-negative (HR‒)/human epidermal growth factor receptor 2-positive (HER2+) subtype (Fig. 1f). The most frequently occurring fusion genes in each subtype were *TTC6::MIPOL1* in HR+/HER2‒ breast cancers, *LHFPL5::CLPSL1* in triple-negative breast cancer (TNBC), and *TPTE2::MRPS31P2* in HER2+ breast cancers (Fig. 1g). We further explored the relationship between fusion genes and prognosis across different subtypes. Specifically, we found that fusion genes were associated with shorter overall survival (OS), recurrence-free survival (RFS), and distant metastasis-free survival (DMFS) only in the HR+/HER2‒ subtype. No such associations were observed for the other subtypes (Fig. 1h). These data suggest the potential clinical relevance of fusion genes in HR+/HER2‒ breast cancer, warranting further functional investigations.

### Dissecting features influencing the landscape of gene fusions in HR+/HER2‒ breast cancer

**Fig. 2:**
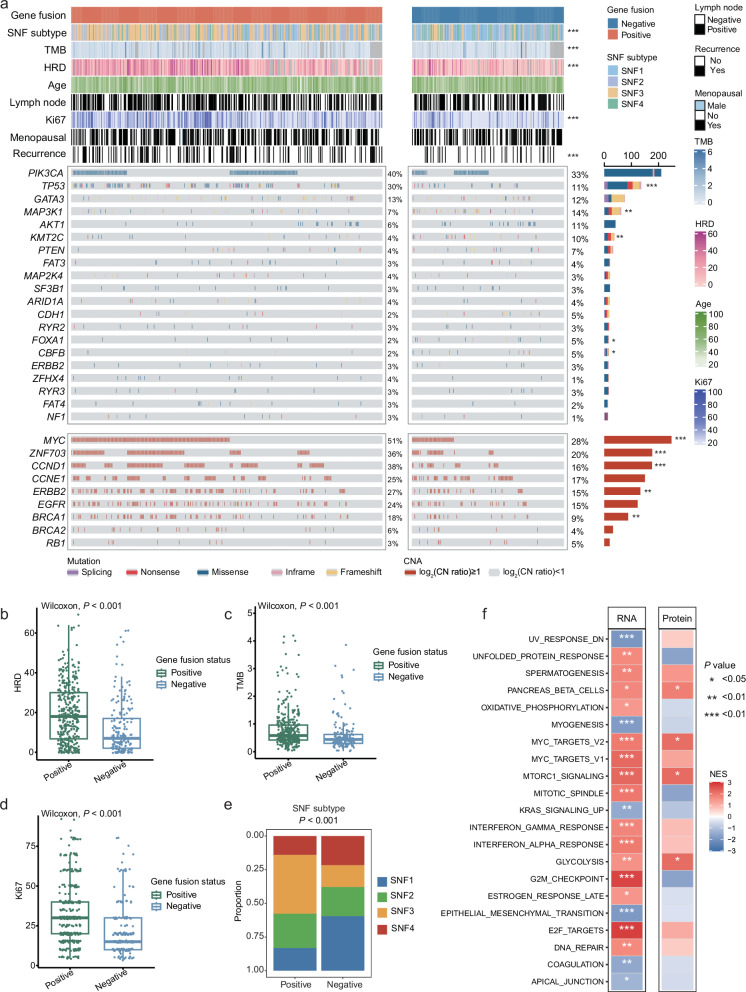
Genomic landscape of fusion genes in HR+/HER2‒ breast cancer. **a** HR+/HER2− breast cancer samples with mutation and/or copy number alteration data are ordered by fusion gene subtype and mutation profile, with clinical and molecular features annotation. Asterisks indicate associations with HR+/HER2‒ breast cancer similarity network fusion (SNF) subtypes, lymph node status, menopausal status, recurrence status and somatic copy number alterations, which were tested with Pearson’s chi-square test; TMB, age, Ki67 and HRD score were tested with the Kruskal–Wallis test; and somatic mutations were tested with Fisher’s exact test. ***P < 0.001, **P < 0.01, *P < 0.05. **b**−**e** Distribution of the HRD score (**b**), TMB score (**c**), Ki67 index (**d**), and HR+/HER2‒ breast cancer similarity network fusion (SNF) subtypes (**e**) across the fusion gene subtypes in HR+/HER2‒ breast cancer. **f** GSEA results showing the top 20 enriched HALLMARK gene sets in fusion gene-positive tumors compared with fusion gene-negative tumors at the RNA and protein level.

To gain insights into the genetic causes of the stronger association between fusion genes and prognosis in HR+/HER2‒ breast cancer than in other subtypes, we looked for associations between fusion genes and driver mutations in cancer-related genes. We found that fusion genes were associated with a higher mutation frequency in several genes, notably *TP53* (29.6% in fusion-positive tumors vs 11.3% in fusion-negative tumors, *P* < 0.001) (Fig. 2a). This finding supports previous analyses indicating that *TP53* plays a pivotal role in maintaining genomic stability^17^, with *TP53* mutations promoting genomic rearrangements^18,19^. We also observed correlations between the presence of fusion genes and increased tumor mutation burden (TMB), an elevated Ki67 index, and higher homologous recombination deficiency (HRD) scores (Fig. 2b‒d). Additionally, these patients had a greater proportion of subtypes characterized by high proliferation and genomic instability^20^ (Fig. 2e). Consistently, gene set enrichment analysis (GSEA) of differentially expressed genes and protein abundance revealed significant enrichment of HALLMARK gene sets associated with DNA damage repair, cell cycle regulation, and inflammatory responses in tumor samples harboring fusion genes (Fig. 2f). Notably, nucleotides were significantly upregulated in fusion-positive tumors, indicating an increased DNA replication and repair activity (Supplementary Fig. S1a). Some lipids, particularly glycerophospholipids, were enriched in fusion-positive tumors, suggesting frequent cell membrane renewal (Supplementary Fig. S1b). Furthermore, we conducted differential abundance analysis between fusion-positive and fusion-negative tissues and determined whether these metabolites were from the Kyoto Encyclopedia of Genes and Genomes (KEGG) metabolic pathways^21^. Our findings consistently revealed high differential abundance scores in metabolic pathways related to glycerophospholipids, amino acids and purines in fusion-positive samples (Supplementary Fig. S1c). Collectively, the presence of fusion genes may be associated with genomic instability and active DNA replication in HR+/HER2‒ breast cancer, which may, in part, explain the correlation between fusion genes and poor prognosis.

Genome-wide analysis of the distribution of somatic fusion genes across the cancer genome has revealed considerable variation in the incidence of fusion genes. Rearrangement hotspots were observed on +8q, +11q, and +17q, frequently involving both intrachromosomal and interchromosomal rearrangements (Supplementary Fig. S2a, bottom). These regions also coincided with high level of copy number amplification, where focal amplifications (greater than 4×) were identified, and frequently included oncogenes or master transcription regulators, including *ERBB2*, *CCND1*, *ZNF703*, and *MYC* (Supplementary Fig. S2a, top). Additionally, we observed that in breast cancers with translocations between 11q and 17q, these regions often harbored copy number amplifications of 11q and 17q (Supplementary Fig. S2b). Similar patterns of copy number alterations and rearrangements were also observed in cases with translocations between 11q and 8q (Supplementary Fig. S2c). In summary, focal amplifications observed in HR+/HER2‒ breast cancer often occur at the boundaries of interchromosomal translocations and are frequently accompanied by oncogene amplification.

### Characteristics of *ADK* fusion genes in HR+/HER2‒ breast cancer

**Fig. 3:**
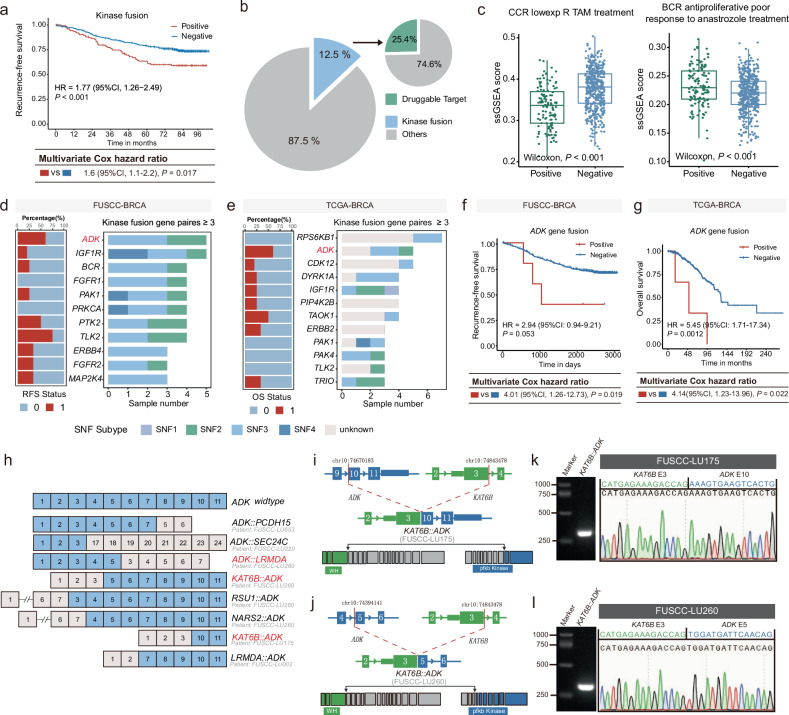
Clinicopathologic characteristics of kinase fusion-positive HR+/HER2– breast cancer samples. **a** Kaplan–Meier analysis of RFS in patients who were kinase fusion gene positive or negative. *P* values calculated with the Mantel–Cox log-rank test. A multivariate Cox proportional hazards model was used to obtain hazard ratios and *P* values, adjusting for the confounders of age, histology, T stage and N stage. **b** The percentage of kinase gene fusions (blue) detected in all gene fusions as well as the percentage of kinase fusions validated as druggable targets (green) in these subsets. **c** Comparison of tamoxifen and anastrozole response scores between groups. Two-sided *P* values were calculated with the Wilcoxon test. **d**, **e** Frequencies of kinase fusions in different subtypes of FUSCC (**d**) and TCGA (**e**) HR+/HER2‒ breast cancer are presented on the right side (various colors represent different subtypes). The survival status-specific distribution of these genes is presented on the left side (various colors represent different survival statuses). **f** Kaplan–Meier analysis of RFS in patients who were positive or negative for fusion genes. *P* values were calculated by the Mantel–Cox log-rank test. A multivariate Cox proportional hazards model was used to obtain hazard ratios and *P* values, adjusting for confounders of age and T stage. **g** Kaplan–Meier analysis of OS in patients who were positive or negative for *ADK* fusion genes. *P* values were calculated with the Mantel–Cox log-rank test. A multivariate Cox proportional hazards model was used to obtain hazard ratios and *P* values, adjusting for the confounders of age, T stage, N stage, and M stage. **h** Exon composition comparing full-length, wild-type *ADK* with fusions identified in FUSCC HR+/HER2‒ breast tumor samples. Five patient samples harbored seven *ADK* fusions. The red annotation indicates in-frame fusion. **i**, **j** Schematics of the genomic structure and mRNA transcripts of the *KAT6B::ADK* fusion genes in FUSCC-LU175 (**i**) and FUSCC-LU260 (**j**) breast cancer samples. Breakpoints in both genes are indicated by linked arrows. The blue bar indicates exons coding for the Pfkb domains. The green bar indicates exons coding for the WH domains. **k**, **l** RT-PCR analyses of *KAT6B::ADK* fusion in FUSCC-LU175 (**k**) and FUSCC-LU260 (**l**) breast cancer samples. Chromatograms show the junction sequences of *KAT6B::ADK* fusion variants detected in FUSCC-LU175 and FUSCC-LU260 tumors.

Fusions involving kinases have been extensively documented as an important class of gene fusions^22,23^ and are especially interesting due to their susceptibility to kinase inhibitors. Our results demonstrate that kinase fusion genes are most frequently observed in the HER2+ breast cancer subtype (Supplementary Fig. S3a). Specifically, the most prevalent kinase fusion genes differed by subtype: *MED1::CDK12* in HER2+ breast cancer, *PTK2::AGO2* in TNBC, and *KAT6B::ADK* in HR+/HER2‒ breast cancer (Supplementary Fig. S3b). Prognostic analysis revealed that kinase fusion positivity is associated with poorer outcomes exclusively in HR+/HER2‒ breast cancer, with no significant prognostic impact observed in other subtypes (Fig. 3a and Supplementary Fig. S3c). We further focused on the characteristics of kinase fusion genes in HR+/HER2‒ breast cancer. Kinase fusion-positive patients accounted for 12.5% of all fusion gene-positive patients, and 25.4% of these fusion genes were targetable by existing drugs (Fig. 3b). The tamoxifen response score decreased in kinase fusion-positive patients, whereas the anastrozole resistance score increased (Fig. 3c). Collectively, these data suggest an association between kinase fusions and resistance to endocrine therapy.

We constructed a comprehensive atlas of the clinicopathological and molecular characteristics of the breast cancer cohort and HR+/HER2‒ breast cancer subtype. Clinically, kinase fusion-positive tumors exhibit higher Ki67 proliferation indices and a greater incidence of lymph node metastasis, suggesting an association with more aggressive disease (Supplementary Figs. S3d, S4). At the molecular level, we observed a reduced frequency of *MAP3K1* mutations in kinase fusion-positive cases compared with that in fusion-negative cases, both in the overall cohort and in HR+/HER2‒ breast cancer (Supplementary Figs. S3d, S4). Given that *MAP3K1* is a serine/threonine kinase involved in multiple signaling pathways and that its loss-of-function mutations are known to drive tumorigenesis^24^, our findings suggest that kinase fusions may represent an alternative mechanism for kinase pathway activation in breast cancer, independent of mutational events.

We then analyzed the frequency of kinase fusion genes in HR+/HER2‒ breast cancer. In both the FUSCC-BRCA cohort and the TCGA-BRCA cohort, *ADK* fusions were highly prevalent (Fig. 3d, e). Notably, we observed a worse prognosis for patients with *ADK* fusions (Fig. 3f, g). However, no association with prognosis was observed for other frequently occurring kinase fusions, such as *IGF1R* (Supplementary Fig. S5a, b). We also observed that patients with *ADK* fusions had higher *ADK* mRNA expression levels (Supplementary Fig. S5c). Owing to sample size limitations, no significant differences were observed in the proteomic levels or copy number scores (Supplementary Fig. S5d, e). Moreover, our analysis revealed that tumors with *ADK* fusion genes tended to be larger (Supplementary Fig. S5f). In summary, *ADK* fusions are the most common partner kinase genes in HR+/HER2‒ breast cancer and are closely associated with worse prognosis. Accordingly, we proceeded to conduct an in-depth investigation into the biological functions of *ADK* fusion genes in the subsequent stages of our research.

To elucidate the intricate roles of *ADK* fusion genes in HR+/HER2‒ breast cancer, we identified several distinct fusion events involving *ADK* with 6 different partner genes: *PCDH15*, *SEC24C*, *KAT6B*, *RSU1*, *NARS2*, and *LRMDA* (Fig. 3h). Notably, the *KAT6B::ADK* fusion was detected in two independent patients, with both patients exhibiting in-frame fusion transcripts. *KAT6B::ADK* also ranked among the top fusion events according to read count in the FUSCC-LU175 and FUSCC-LU260 tumor samples (Supplementary Fig. S5g). These collective findings suggest the potential functional significance of this recurrent fusion. The fusion site of *KAT6B* was mapped to exon 3, whereas the fusion sites of *ADK* were identified in exons 10 and 5. The fused KAT6B retained its winged helix (WH) domain, whereas ADK preserved part of its carbohydrate kinase PfkB domain (Fig. 3i, j). We successfully amplified the genomic fusion points in *KAT6B::ADK* positive tumors from the FUSCC-BRCA cohort (Fig. 3k, l). Sanger sequencing confirmed breakpoint junctions in the genomic DNA, leading us to designate the *KAT6B::ADK* fusion genes found in the FUSCC-LU175 and FUSCC-LU260 breast cancer samples as *KAT6B::ADK*(175) and *KAT6B::ADK*(260), respectively.

### *KAT6B::ADK* promotes tumor metastasis and endocrine therapy resistance

**Fig. 4:**
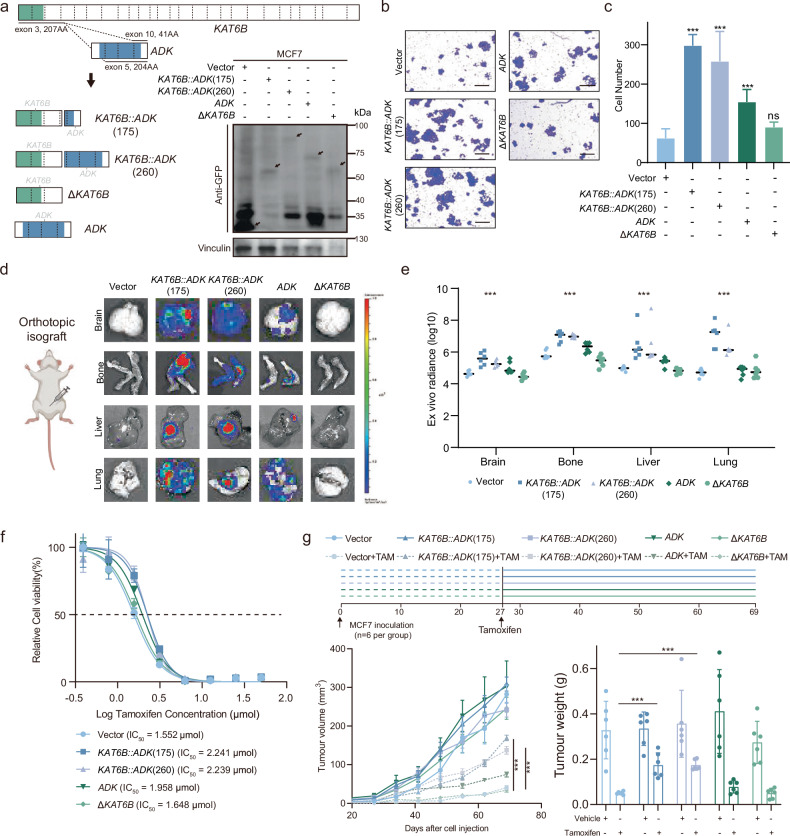
*KAT6B::ADK* increased cell migration, metastasis, and tamoxifen resistance. **a** Diagrammatic representation of the *KAT6B*-N-term (Δ*KAT6B*), *ADK* wild type (*ADK*) and *KAT6B::ADK* fusions in the FUSCC-LU175 (*KAT6B::ADK*(175)) and FUSCC-LU260 (*KAT6B::ADK*(260)) samples; constructs were expressed in MCF7 cells and validated by immunoblotting for GFP. **b**, **c** Transwell assays were performed to evaluate the effects of vector, *KAT6B::ADK*, *ADK,* and Δ*KAT6B* on the migration of MCF7 cells. Representative images (**b**) and quantification of relative migrated cells (**c**) are shown (mean ± SD; *n* = 3 biological replicates). Two-tailed Student’s *t*-test. Scale bars, 100 μm. **d** Representative ex vivo luminescence images showing MCF7 cells in the lungs, brain, liver, and bones of mice subjected to vector, *KAT6B::ADK*, *ADK*, and Δ*KAT6B* overexpression. Scale bar, 1.5 cm. **e** Ex vivo bioluminescence imaging of metastatic cells in various organs was quantified and compared across experimental groups. **f** MCF7 cells were treated for 48 h with increasing concentrations of tamoxifen, and their viability was measured using a CCK-8 assay. The data are presented as the means ± SD from three independent experiments. **g** MCF7 cells infected with lentivirus encoding control, *KAT6B::ADK*, *ADK*, or Δ*KAT6B* were injected orthotopically into the mammary fat pads of 6-week-old NOD/SCID female mice, with six mice per group. Each group was treated with tamoxifen or vehicle. After 70 days, the tumors were collected. Tumor volume and weight were measured. The data are presented as the mean ± SEM. Two-tailed Student’s *t*-test. **P < 0.01; ***P < 0.001.

To elucidate the biological impact and mechanism of *KAT6B::ADK* in HR+/HER2‒ breast cancer progression, we initially tested the presence of *KAT6B::ADK* fusion genes in HR+/HER2‒ cell lines. The fusion was absent in both MCF7 and T47D cells (Supplementary Fig. S6a). We then introduced the full-length open-reading frames of *KAT6B::ADK* into the fusion-negative breast epithelial cell lines MCF7 and T47D. Cells transduced with a vector containing truncated *KAT6B* (Δ*KAT6B)* or wild-type *ADK* were used as controls (Fig. 4a and Supplementary Fig. S6b). Ectopic expression of *ADK* was used to mimic the overexpression resulting from *ADK* gene amplification.

We next assessed the role of *KAT6B::ADK* fusions in cell migration and invasion. We observed a significant increase in cell migration and invasion in cells with ectopic expression of *KAT6B::ADK*, compared with vector control and *ADK*-expressing cells (Fig. 4b, c and Supplementary Fig. S6c, d). When the proliferative activity driven by the *KAT6B::ADK* fusions was evaluated, only a modest increase in cell proliferation was detected in HR+/HER2‒ breast cancer cells (Supplementary Fig. S6e‒g). Further in vivo studies using orthotopic xenograft models confirmed the prometastatic effect of *KAT6B::ADK*, which promoted the metastasis of MCF7 cells to distant organs, including the lungs, brain, liver, and bones (Fig. 4d, e). Collectively, these findings underscore the role of *KAT6B::ADK* in enhancing the migratory and metastatic potential of HR+/HER2‒ breast cancer cells.

Anti-estrogen endocrine therapy is the primary treatment for HR+/HER2‒ breast cancer. Given our previous observations linking *ADK* fusion genes positivity to shorter survival in HR+/HER2‒ breast cancer patients, we hypothesized that *KAT6B::ADK* may contribute to endocrine therapy resistance. RNA sequencing revealed an increase in single-sample GSEA scores associated with endocrine therapy resistance in MCF7 cells overexpressing *KAT6B::ADK* and *ADK* (Supplementary Fig. S6h, i). Importantly, these findings were clinically corroborated in the FUSCC-BRCA cohort, in which *ADK* fusions-positive tumors similarly had elevated endocrine therapy resistance scores (Supplementary Fig. 6j, k). Tamoxifen sensitivity assays in the MCF7 and T47D cell lines revealed an increase in the tamoxifen IC_50_ upon overexpression of *KAT6B::ADK*, indicating that *KAT6B::ADK* promotes tamoxifen resistance (Fig. 4f and Supplementary Fig. S6l‒n). Consistent results were observed in the colony formation assays following tamoxifen treatment (Supplementary Fig. S6o). To further validate this finding, we treated tumor-bearing mice with ectopic expression of *KAT6B::ADK* and *ADK* with tamoxifen in vivo, and tumors overexpressing *KAT6B::ADK* showed a minimal response to tamoxifen treatment, in contrast to the significant tumor inhibition in the control group (Fig. 4g and Supplementary Fig. S6p). These results collectively indicate that *KAT6B::ADK* is implicated in endocrine therapy resistance in HR+/HER2‒ breast cancer, thereby facilitating tumor progression.

On the basis of our finding of enhanced cell migration, we explored the oncogenic potential of *KAT6B::ADK*. Ectopic expression of *KAT6B::ADK* in NIH-3T3 cells significantly promoted proliferation in vitro and induced tumorigenesis in immunodeficient mice (Supplementary Fig. S7a‒f), confirming its oncogenic capacity^25^. Similarly, introducing *KAT6B::ADK* into MCF10A cells disrupted acinar morphology under three dimensional (3D) basement membrane culture^15^, leading to increased numbers of filled acinar structures and the emergence of large, disordered structures (Supplementary Fig. S7g‒i). These changes further support the oncogenic potential of *KAT6B::ADK* in mammary epithelial cells.

### *KAT6B::ADK* fusion proteins form liquid-like cytoplasmic condensates that activate kinase activity

**Fig. 5:**
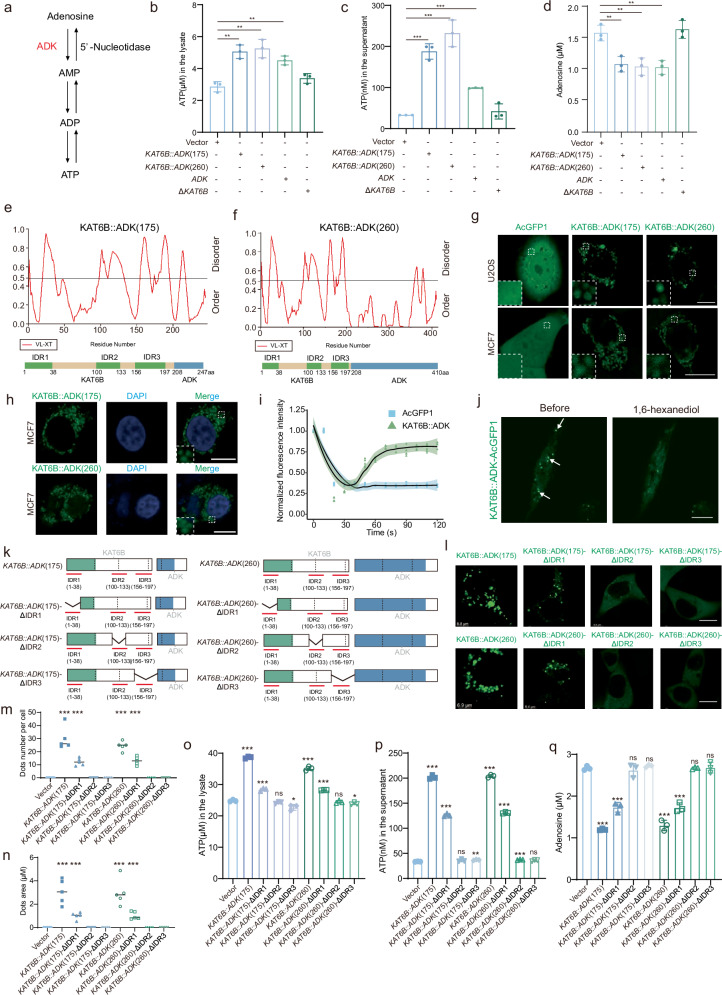
KAT6B::ADK forms liquid–liquid phase separated structures that activate ADK kinase activity. **a** Schematic diagrams of adenosine metabolism processes. **b**, **c** Effects of *KAT6B:ADK* and *ADK* overexpression on the production (**b**) and release (**c**) of ATP in MCF7 cells (*n* = 3). **d** ELISA analysis of intracellular adenosine levels in vector-, *KAT6B::ADK-*, *ADK-*, and *ΔKAT6B-*overexpression MCF7 cells. **e**, **f** *KAT6B::ADK* IDRs were predicted by predictor of natural disordered regions (PONDR; http://pondr.com/). **g** Representative live-cell images of U2OS and MCF7 cells stably expressing KAT6B::ADK fusion proteins tagged with AcGFP1. Scale bar: 10 μm. **h** A representative image of MCF7 cells stably expressing KAT6B::ADK-AcGFP1 merged with DAPI staining. Scale bar: 10 μm. **i** Quantification of the FRAP assay for condensates is shown. Data represent *n* = 5 from three independent experiments. **j** Live imaging shows MCF7 cells stably expressing KAT6B::ADK-AcGFP1 treated with 2.5% 1,6-hexanediol. The arrows indicate representative KAT6B::ADK-AcGFP1 puncta to be dissolved by 1,6-hexanediol. Scale bar, 10 μm. **k** Schematic representation of the domain architecture of the KAT6B::ADK mutants utilized in this study. **l** Representative live-cell images of AcGFP1-tagged KAT6B::ADK and their mutants in U2OS cells. Scale bar: 10 μm. **m**, **n** Quantitative analysis of the biomolecular condensates of KAT6B::ADK and their mutants. *n* = 5 biologically independent cells were pooled from three independent replicates. *P* values determined by two-tailed Student’s *t*-test. **o**, **p** Effects of *KAT6B::ADK* and their different mutants on the production and release of ATP in MCF7 cells. *n* = 3 biological replicates. **q** ELISA analysis of intracellular adenosine levels in MCF7 cells transfected with *KAT6B::ADK* and different mutants. *n* = 3 biological replicates.

Previous studies have identified kinase fusion partner genes that are capable of significantly activating downstream kinase signaling pathways^1^. We hypothesized that KAT6B::ADK might similarly activate ADK kinase activity. Given the established role of *ADK* in ATP production and release^26^, we measured the intracellular and extracellular ATP levels in cells overexpressing *KAT6B::ADK* and *ADK* (Fig. 5a). Our results revealed elevated ATP levels in cells expressing *KAT6B::ADK*, indicating the constitutive activation of ADK kinase activity (Fig. 5b, c). ELISA testing confirmed a reduction in intracellular adenosine levels upon *KAT6B::ADK* and *ADK* expression, further supporting the activation of ADK kinase activity (Fig. 5d). Notably, compared with *ADK* alone, *KAT6B::ADK* induced higher levels of ATP production and release, highlighting the potentiating activation effect of the fusion on ADK activity.

Adenosine dialdehyde treatment of cell lines resulted in a decrease in ADK substrate adenosine levels^27^. Furthermore, adenosine dialdehyde treatment led to a reduction in intracellular ATP levels (Supplementary Fig. S8a, b). Concurrently, adenosine dialdehyde treatment significantly inhibited the promigratory activity of both *KAT6B::ADK* and *ADK* (Supplementary Fig. S8c, d). These findings confirm that the promigratory effects of *KAT6B::ADK* are dependent on the activation of ADK kinase activity. Finally, RNA sequencing analysis of cell lines overexpressing *KAT6B::ADK* and *ADK* revealed the activation of adenosine metabolism-related pathways in both cell lines (Supplementary Fig. S8e). These findings were clinically corroborated in the FUSCC-BRCA cohort, where *ADK* fusions-positive tumors similarly showed activation of adenosine metabolism pathways (Supplementary Fig. S8f). In summary, these studies support the conclusion that *KAT6B::ADK* fusions significantly activate ADK kinase activity and promote the progression of HR+/HER2‒ tumors.

The oligomerization capacity obtained is thought to be required for the downstream activation of kinase fusion oncoproteins. However, this hypothesis cannot explain the oncogenic activation of *KAT6B::ADK* fusions, which lack known oligomerization domains (Fig. 3k, l). Recent studies have investigated the relationship between liquid–liquid phase separation (LLPS) and fusion oncoproteins^28–30^. Therefore, we investigated whether LLPS was involved in the activation of *KAT6B::ADK* fusion genes. We identified three intrinsically disordered regions (IDRs) within the KAT6B moiety of the fusion protein that were predicted to facilitate phase separation (Fig. 5e, f). These include an N-terminal IDR (amino acids 1‒38), referred to as IDR1; a central region in ΔKAT6B (amino acids 100‒133), referred to as IDR2; and a C-terminal region in ΔKAT6B (amino acids 156‒197), referred to as IDR3.

To explore whether the 5′ region replacement of KAT6B in ADK proteins would affect their cellular distribution, we generated U2OS and MCF7 cells that stably expressed C-terminal AcGFP1-tagged KAT6B::ADK fusion proteins. Remarkably, KAT6B::ADK fusions displayed a discrete punctate distribution localized in the cytoplasm (Fig. 5g, h and Supplementary Fig. S9a). Additionally, the ΔKAT6B sequence resulted in the formation of high-density puncta within the nucleus but displayed a diffuse distribution in the cytoplasm. In contrast, ADK proteins were predominantly localized in the nucleus and did not form puncta (Supplementary Fig. S9b). These findings underscore that the N-terminal fusion partner ΔKAT6B altered the subcellular localization and promoted condensates formation of ADK chimeric proteins. Live-cell imaging and fluorescence recovery after photobleaching (FRAP) experiments on KAT6B::ADK-AcGFP1 droplets demonstrated their dynamic nature and liquid-like characteristics, confirming the formation of biomolecular condensates (Fig. 5i). Treatment with 1,6-hexandiol, which is known to disrupt hydrophobic interactions^31^, effectively dissolved these condensates, providing further evidence for their LLPS nature (Fig. 5j).

To elucidate the contribution of individual IDRs to LLPS, we generated KAT6B::ADK variants with individual deletions in IDR1, IDR2, and IDR3 (Fig. 5k). Deletion of IDR1 resulted in fewer and smaller condensates, whereas knockout of IDR2 or IDR3 led to diffuse localization, highlighting the importance of these IDRs in mediating LLPS (Fig. 5l–n). Metabolic changes in ADK pathways in *KAT6B::ADK* mutants with IDR deletions revealed a reduction in ATP production and release, along with an increase in adenosine levels, the ADK metabolic substrate (Fig. 5o‒q). Importantly, these mutant variants restored tamoxifen sensitivity and significantly impaired cell migration (Supplementary Fig. S9c, d). Additionally, cycloheximide chase assays confirmed that KAT6B::ADK and all IDR deletion mutants exhibit comparable protein half-lives, excluding potential effects of IDR sequence deletion on KAT6B::ADK fusion protein stability (Supplementary Fig. S9e). Collectively, our findings establish that KAT6B::ADK fusion proteins form liquid-like condensates through specific IDR domains and that this phase separation is critical for both kinase activation and subsequent oncogenic transformation.

### Systematic classification of *ADK* fusion variants reveals structure-dependent functional properties

To resolve functional heterogeneity among *ADK* fusion variants, we classified these oncoproteins into three categories through integrated structural and functional profiling. The in-frame *ADK::LRMDA* fusion, characterized by preserved *ADK* frame and *LRMDA*-derived IDRs, represents the most potent oncogenic class, exhibiting robust cytoplasmic condensate formation via LLPS, enhanced kinase activity, and strong induction of tamoxifen resistance and cell migration (Supplementary Fig. S10a‒c, j‒o). In contrast, the out-of-frame *ADK::PCDH15* variant retains catalytic competence through N-terminal positioning, preserving the *ADK* frame despite *PCDH15* truncation, manifesting diffuse localization without condensates and maintaining kinase activity levels comparable to those of wild-type *ADK* (Supplementary Fig. S10d‒f, j‒m). The results of functional assays revealed that *ADK::PCDH15* significantly increased both tamoxifen resistance and cell migration capacity but was attenuated compared to *ADK::LRMDA* fusion (Supplementary Fig. S10n, o). Conversely, C-terminal *LRMDA::ADK* fusions with frameshift-induced *ADK* frame ablation showed negligible kinase activity and demonstrated no significant effects on tamoxifen resistance or cell migration, suggesting that these kinase-deficient variants are likely to represent passenger alterations (Supplementary Fig. S10g‒o). These comprehensive analyses revealed two critical requirements for functional *ADK* fusions: (1) preservation of a kinase domain and (2) either IDR-mediated LLPS inducing robust kinase hyperactivation (as in *ADK::LRMDA* and *KAT6B::ADK*) or maintaining enzymatic activity (as in *ADK::PCDH15*). These results established that the functional consequences of *ADK* fusions are critically dependent on the structural features of the fusion partners.

### *KAT6B::ADK* induces an integrated stress response via the GCN2-eIF2α axis

**Fig. 6:**
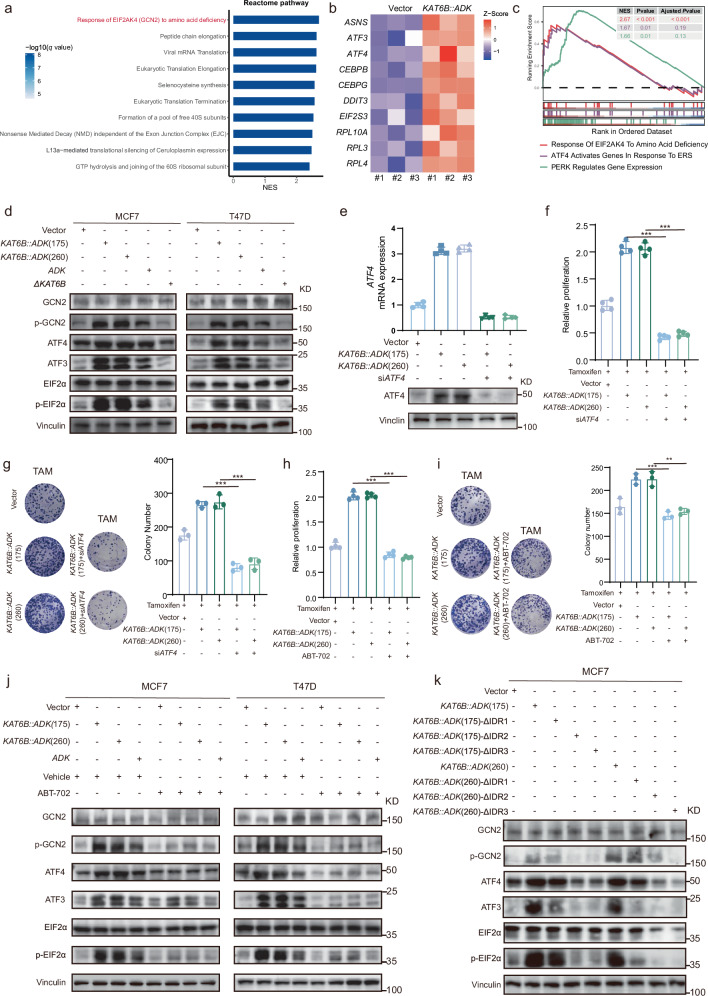
*KAT6B::ADK* induces an ISR by activating ADK kinase activity. **a**, **b** Reactome pathway analysis (**a**) and expression of genes enriched in the “Response of EIF2AK4 (*GCN2*) to amino acid deficiency” pathway (**b**) in *KAT6B::ADK*-overexpression MCF7 cells. NES, normalized enrichment scores. **c** GSEA revealed upregulation of ISR-related pathways in *KAT6B::ADK*-overexpressing MCF7 cells. **d** Levels of the indicated molecules in the indicated cells transfected with vector, *KAT6B::ADK*, *ADK*, and Δ*KAT6B*. A representative western blot result from three independent experiments is shown. **e** Validation of the mRNA and protein levels of ATF4 in MCF7 cells transfected with siNC or si*ATF4*. **f**, **g** MCF7 cells stably expressing the indicated constructs and transfected with si*ATF4* were subjected to growth analysis (*n* = 4) (**f**) or colony formation analysis (**g**). **h**, **i** MCF7 cells stably expressing the indicated constructs and treated with GCN2i were subjected to growth analysis (*n* = 4) (**h**) or colony formation analysis (**i**). **j** Levels of the indicated molecules in MCF7 cells transfected with vector, *KAT6B::ADK*, and *ADK* and treated with ABT-702. **k** Levels of the indicated molecules in MCF7 cells transfected with *KAT6B::ADK* and their different mutants.

We next investigated the mechanism by which *KAT6B::ADK* activates kinase activity, hence promoting breast cancer cell migration and endocrine therapy resistance. Utilizing GSEA with the Reactome database, we observed a pronounced upregulation of the integrated stress response (ISR) signaling pathway in MCF7 cells overexpressing *KAT6B::ADK* (Fig. 6a). This result was further supported by the elevated expression of the core transcription factors ATF4 and ATF3, which are integral to the ISR (Fig. 6b). Additionally, enrichment of other ISR-related pathways was noted, underscoring the activation of ISR by *KAT6B::ADK* (Fig. 6c).

Activation of the ISR is closely linked to the phosphorylation of eIF2α, a key regulatory step in the pathway^32^. In both MCF7 and T47D cells overexpressing *KAT6B::ADK*, we detected increased levels of ATF4, ATF3, phosphorylated eIF2α (p-eIF2α), and phosphorylated GCN2 (p-GCN2), along with elevated mRNA expression of *ATF3* and *ATF4* (Fig. 6d and Supplementary Fig. S11a, b). Both *ATF4* knockdown and GCN2 inhibition reversed tamoxifen resistance, establishing the ISR pathway as the mechanistic link between *KAT6B::ADK* and therapeutic resistance (Fig. 6e‒i). Notably, treatment with the ADK-specific inhibitor ABT-702 effectively abrogated *KAT6B::ADK*-mediated upregulation of these factors, implicating the ISR pathway in response to *KAT6B::ADK* dependence on ADK kinase activation (Fig. 6j). Moreover, this upregulation was reversed by deletion of the IDR sequences, demonstrating the requirement for KAT6B::ADK fusion proteins to form liquid-like condensates in activating ISR via the GCN2-eIF2α axis (Fig. 6k). These findings suggest that *KAT6B::ADK* fusion genes modulate cellular stress responses, thus potentially contributing to the adaptability and survival of breast cancer cells and driving tamoxifen resistance.

### Therapeutic potential of ADK inhibition in patients with *KAT6B::ADK* fusion genes

**Fig. 7:**
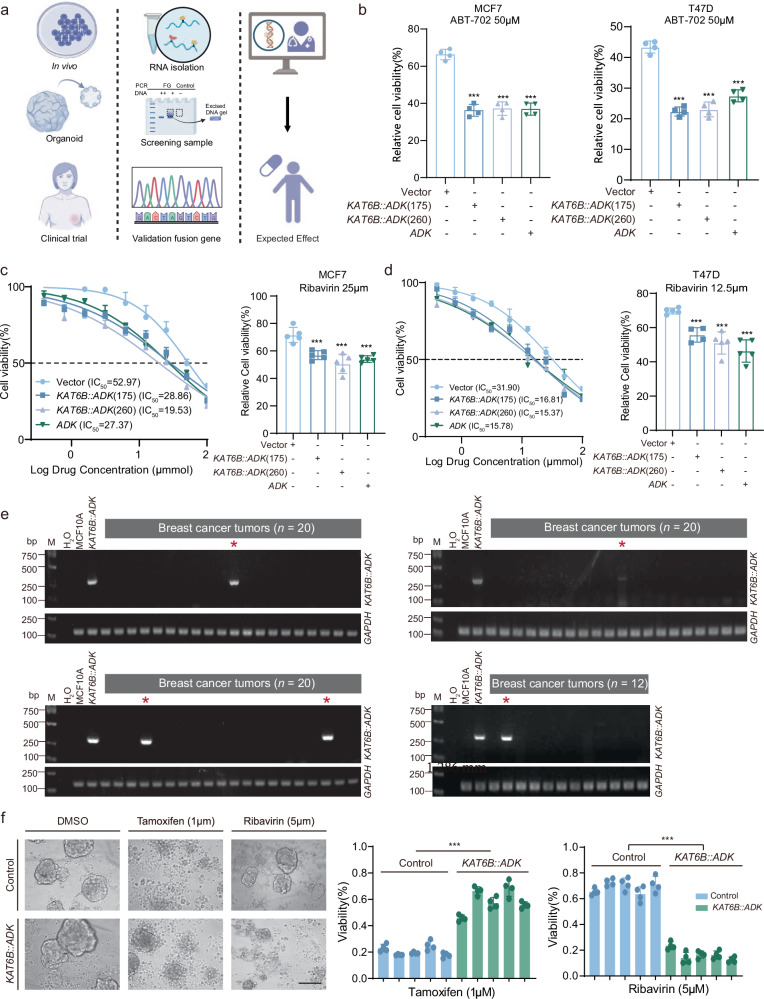
Therapeutic response of tumors with *KAT6B::ADK* fusions to *ADK* inhibitor treatment. **a** Schematic overview of the study design. **b** Viability assays of inhibitor-treated cells. MCF7 and T47D cells were treated for 48 h with 50 μM ABT-702, and cell viability was measured using a CCK-8 assay. The data are presented as mean ± SD from three independent experiments. **c**, **d** Dose−response curves after 48 h of drug treatment with ribavirin in MCF7 **c** and T47D **d** cells expressing *KAT6B::ADK*, *ADK*, Δ*KAT6B,* and vector. Cell viability was normalized to that of the vehicle (DMSO)-treated cells. **e** RT-PCR analysis of *KAT6B::ADK* fusion in HR+/HER2‒ tumors from the FUSCC-PDOs cohort is shown. MCF10A cells were used as a negative control. cDNA derived from breast cancer samples expressing the *KAT6B::ADK* fusion was used as a positive control. *GAPDH* transcripts were used as an internal loading control. M: DNA ladder marker. **f** Representative images of PDOs with or without *KAT6B::ADK* fusion (left) and the viability of PDOs following treatment with tamoxifen and ribavirin (right). The data are presented as the mean ± SD. Scale bar: 200 μm. *P* values were obtained from Student’s *t*-test. ***P < 0.001.

ADK primarily serves as a therapeutic target for conditions such as epilepsy, pain syndromes, and inflammation, along with several inhibitors, such as adenosine^33^ and ribavirin^34^, which are widely used in clinical practice. Recently, accumulating evidence has supported *ADK* as a therapeutic target for cancer^35,36^. Given the established role of *KAT6B::ADK* in promoting endocrine therapy resistance and its ability to activate ADK kinase activity, we explored the therapeutic potential of ADK inhibition in patients harboring *KAT6B::ADK* fusions. We employed various experimental models, including in vitro cell lines and PDOs, to perform multidimensional functional validation and explore drug sensitivity in tumors with *KAT6B::ADK* fusion events (Fig. 7a).

Our in vitro studies demonstrated that HR+/HER2‒ cell lines expressing *KAT6B::ADK* were sensitized to the ADK-specific inhibitor ABT-702, as well as to ribavirin, a broad-spectrum antiviral agent known to inhibit ADK^34^ (Fig. 7b‒d). These findings suggest that ADK inhibition may reverse the endocrine resistance phenotype conferred by *KAT6B::ADK* fusions. To ascertain the clinical relevance of our findings, we conducted genomic PCR on a cohort of 192 breast cancer tumor samples from patients of Fudan University Shanghai Cancer Center (FUSCC), and identified *KAT6B::ADK* fusion variants in five cases (Fig. 7e and Supplementary Fig. S12a). Sanger sequencing of the fusion junctions in PDOs confirmed that the breakpoints were identical to those in the FUSCC-BRCA cohort (Supplementary Fig. S12b). Notably, ADK protein expression was elevated in *KAT6B::ADK* fusion-positive PDOs compared with that in fusion-negative PDOs (Supplementary Fig. S12c). Importantly, PDOs harboring *KAT6B::ADK* fusions exhibited increased sensitivity to ADK inhibitors, as evidenced by a significant reduction in viability upon treatment with ribavirin (Fig. 7f). These PDOs also displayed the characteristics of tamoxifen resistance (Fig. 7f). Overall, these data support the use of ADK inhibitors as a promising treatment strategy for HR+/HER2‒ patients with *ADK* fusions.

**Fig. 8:**
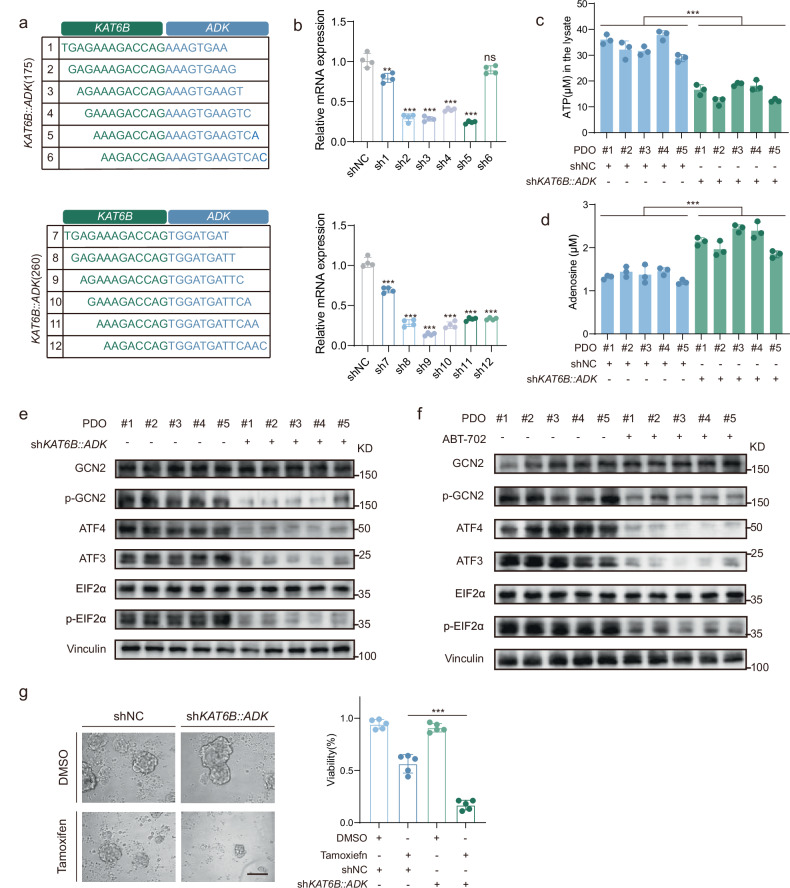
Fusions gene knockdown reverses *KAT6B::ADK*-driven metabolic reprogramming and endocrine resistance in PDOs. **a** Sequences of shRNA tiling over the fusion junction; green and blue letters denote the *KAT6B* and *ADK* sequences, respectively, in *KAT6B::ADK* fusions. **b** Validation of the mRNA levels of *KAT6B::ADK* in PDOs transfected with shNC or sh*KAT6B::ADK*. **c** Effects of shNC or sh*KAT6B::ADK* on the production of ATP in PDOs. *n* = 3 biological replicates. Significance was determined by Student’s *t*-test (**P < 0.01, ***P < 0.001). **d** ELISA analysis of adenosine from PDOs transfected with shNC or sh*KAT6B::ADK*. *n* = 3 biological replicates. Significance was determined by Student’s *t*-test. **e** Protein levels of the indicated molecules in PDOs transfected with shNC or sh*KAT6B::ADK*. **f** Levels of the indicated molecules in PDOs with *KAT6B::ADK* fusions after treatment with DMSO or ABT-702. **g** Representative images of PDOs transfected with shNC or sh*KAT6B::ADK* and the viability of PDOs following treatment with DMSO or tamoxifen. The data are presented as means ± SD. Scale bar, 200 μm. *P* values were obtained from Student’s *t*-test. ***P < 0.001.

Moreover, we developed a targeted shRNA approach against the *KAT6B::ADK* fusion junction to perform multifaceted mechanistic validation (Fig. 8a). In PDOs harboring endogenous *KAT6B::ADK* fusions, this strategy achieved efficient fusion transcript knockdown (Fig. 8b). The knockdown induced significant metabolic reprogramming, including decreased intracellular ATP levels and increased adenosine concentrations (Fig. 8c, d), which was consistent with reduced ADK kinase activity. Molecular analysis revealed that both knockdown of *KAT6B::ADK* and pharmacological inhibition of ADK with ABT-702 attenuated ISR pathway activation (Fig. 8e, f), demonstrating that both the KAT6B::ADK fusion protein and its kinase activity are essential for ISR induction. Moreover, *KAT6B::ADK* knockdown restored tamoxifen sensitivity in PDOs (Fig. 8g). The consistent phenotypic reciprocity between overexpression systems and endogenous knockdown models provides compelling evidence that *KAT6B::ADK*-mediated ADK kinase activation drives ISR pathway activation and endocrine therapy resistance, establishing these findings in physiologically relevant contexts.

## Discussion

To address the current limitations of large-scale and multidimensional studies of annotated fusion genes in breast cancer, we utilized RNA sequencing data from 1361 breast cancer patient samples to identify clinically relevant fusion genes and analyze the clinical and genomic characteristics of patients harboring these fusions. Additionally, we established a PDOs cohort comprising 192 patients to explore clinical translational strategies for fusion genes. Through integrative bioinformatics analysis and functional validation, we identified a novel *KAT6B::ADK* fusion as a promoter of breast cancer metastasis and endocrine therapy resistance. Our findings suggest that *KAT6B::ADK* induces constitutive kinase activation via LLPS and exhibits translational potential, which can be inhibited by ribavirin treatment. Overall, this study provides comprehensive, large-scale, and multidimensional annotations of fusion genes in breast cancer. Our study highlights the potential of fusion genes as true driver events in breast cancer, serving as both predictive biomarkers and targets for therapeutic intervention.

Although several studies have investigated the fusion gene landscape in breast cancer, many of these studies lack comprehensive functional validation and fail to address their therapeutic implications^16,37,38^. Furthermore, these studies are often limited by small sample sizes, single-omics approaches, and the absence of multidimensional sequencing data or validation cohorts to assesses therapeutic efficacy^14,39^. In contrast, our study employs an integrative, multiomics strategy and robust functional analyses to elucidate the biological roles and clinical significance of fusion genes. By leveraging a large breast cancer cohort, our study was uniquely positioned to identify novel predictive biomarkers. The inclusion of extensive prognostic data and comprehensive multiomics integration significantly enhanced the depth and breadth of our findings. Additionally, we systematically evaluated the impact of fusion genes on tumor behavior and their clinical relevance, underscoring their potential as actionable therapeutic targets. Notably, our study establishes a valuable resource for annotated fusion gene data, which provides a foundation for further biological insights and advances in the understanding of breast cancer pathogenesis.

Recent transcriptomic and genomic sequencing studies have revealed oncogenic gene fusions in patients with breast cancer. However, these efforts have not been sufficient to fully characterize the fusion gene landscape or elucidate molecular targets for personalized treatment. For example, *ESR1::CCDC170* has been detected in 6%–8% of Luminal B breast cancers^40,41^, a more aggressive and endocrine-resistant subtype of HR+/HER2‒ breast cancer, and has been shown to promote increased tumor invasiveness and endocrine resistance. Similarly, *BCL2L14::ETV6* enhances tumor invasiveness and taxane resistance in TNBC^14^. However, these studies primarily revealed the biological functions and biomarker potential of fusion genes without advancing their utility as therapeutic targets. In contrast, some studies on fusion genes in breast cancer have focused primarily on kinase fusions with established oncogenic roles in other cancers and known targeted inhibitors. For example, Paratala et al. described the frequency, oncogenic potential, and therapeutic relevance of the RET oncogene in breast cancer^42^, whereas Ross et al. characterized kinase fusions with available targeted therapies using MSK-IMPACT targeted DNA sequencing and MSK-fusion targeted RNA sequencing^39^. These studies, however, largely overlooked other fusion genes present in breast cancer. In this study, we conducted a large-scale cohort analysis to characterize the landscape of fusion genes in breast cancer. We identified *ADK* fusions as the most prevalent kinase fusion gene in HR+/HER2‒ breast cancer, which is closely associated with accelerated disease progression and shorter survival. These findings provide molecular insights into the distinct pathologies of fusion genes and highlight their potential as promising biomarkers and therapeutic targets for breast cancer.

LLPS underlies the formation and function of membrane-less compartments and serves as a fundamental mechanism for regulating various biological processes and disease progression^43^. Studies have shown that abnormal phase separation or the dysfunction of condensates within pathways can disrupt cellular processes, contributing to cancer development^44^. Kinase fusion events often retain kinase activity, leading to constitutive activation and enhanced downstream signaling. This ligand-independent activation is typically attributed to oligomerization or dimerization of the kinase, driven by the oligomerization domains in the fusion partner^1,45^. However, this hypothesis does not account for the oncogenic activation of kinase fusions that lack known oligomerization domains. Recent studies have revealed a relationship between LLPS and fusion oncoproteins, such as *NTRK* fusions^28,29,46^. In this study, we demonstrated that ADK fusion proteins readily form liquid-like condensates, mediated by the upstream fusion partner, KAT6B. These condensates act as reaction hubs for ADK kinase activation and as organizational centers for downstream signal transduction. While our functional data established that IDR deletion disrupts LLPS and oncogenic phenotypes, the precise conformational rearrangements induced by these deletions in the KAT6B::ADK fusion protein remain unresolved. Further structural studies are warranted to elucidate how IDR removal alters the molecular architecture of the fusion protein. Notably, we also identified a distinct class of oncogenic *ADK* fusions (e.g., *ADK::PCDH15*) in which C-terminal truncation preserves kinase activity despite the absence of LLPS capacity. These findings extend recent work demonstrating that out-of-frame fusion events can generate functional, malignancy-promoting truncations^47^. However, in our analyses, LLPS-competent *ADK* fusions (e.g., *ADK::LRMDA* and *KAT6B::ADK*) induce malignant phenotypes more potently than *ADK*-frame preserved but LLPS-incompetent variants do. Overall, our findings highlight that LLPS is essential for the activation of *ADK* fusions, thereby providing new insights into their oncogenic mechanisms.

Targeting *ADK* fusions represents a promising therapeutic strategy for HR+/HER2‒ breast cancer. In our study, we observed that HR+/HER2‒ breast cancer patients harboring *ADK* fusions exhibited shorter RFS and OS as well as resistance to endocrine therapy. Notably, phenotypic studies using a cohort of breast cancer PDOs revealed a synergistic effect when the ADK inhibitor ribavirin was combined with endocrine therapy. Ribavirin is a synthetic guanosine nucleoside analog with broad-spectrum antiviral properties that serves as a substrate for ADK metabolism and effectively inhibits ADK activity^48^. Recently, the efficacy of ribavirin in cancer has been explored in various preclinical models and ongoing clinical trials across cancers such as acute myeloid leukemia, oropharyngeal squamous cell carcinoma, and metastatic breast cancer^49^. Whether ribavirin is effective for patients with *ADK* fusions warrants further investigation in a breast cancer patient cohort. In fact, we plan to conduct large-scale prospective *KAT6B::ADK* screening using the FUSCC precision medicine platform to address this question. Overall, our study provides new insights into risk stratification and optimized targeted therapies for HR+/HER2‒ breast cancer patients.

In our study, *KAT6B::ADK* fusions were detected at differing frequencies between the FUSCC-BRCA cohort (2/563, 0.4%) and the PDOs cohort (5/192, 2.6%). This apparent discrepancy warrants consideration of methodological and biological factors. First, the requirement for larger tumor specimens to establish viable PDOs may have introduced selection bias, as tumors harboring *ADK* fusions tended to be larger (Supplementary Fig. S5f), potentially reflecting increased genomic instability or clonal expansion advantageous to fusion-bearing subclones^15^. Second, differences in technical sensitivity likely contributed: RT-PCR (used for PDO screening) can detect fusions at extremely low frequencies (1 in 10^5^‒10^6^ cells), whereas RNA-seq (used for the FUSCC-BRCA cohort) is inherently limited by sequencing depth and algorithmic thresholds for reliable fusion calling, potentially missing low-abundance events^50–53^. Crucially, Sanger sequencing confirmed identical *KAT6B::ADK* breakpoints in all five PDO cases and the original FUSCC-BRCA patients (Supplementary Fig. 12b), validating the biological authenticity of these fusions. Collectively, these findings underscore that the observed fusion prevalence can be influenced by cohort-specific selection criteria and detection methodologies. Future studies should employ orthogonal validation approaches and account for potential sampling biases when interpreting fusion frequencies.

Although our study provides valuable insights, certain limitations should be acknowledged. The relatively low prevalence of *ADK* fusions in our cohort may limit the generalizability of our findings. Future studies with larger cohorts and more diverse patient populations are warranted to validate the clinical utility of *ADK* fusions and other functional kinase fusion genes as biomarkers and therapeutic targets. Specifically, elevated read counts of kinase fusion genes may serve as a useful preliminary indicator of functional significance. Additionally, the functional implications of other *ADK* fusion variants require further investigation to fully elucidate the spectrum of *ADK*-driven oncogenesis. Finally, large-scale prospective clinical studies are needed to validate and extend our findings, particularly to confirm the therapeutic potential of ribavirin as a novel targeted treatment for patients with *ADK* fusions.

In summary, we utilized a large-scale multiomics cohort and drug-testing platform to investigate fusion genes in breast cancer and elucidate their therapeutic implications. We identified *ADK* fusions as oncogenic drivers in HR+/HER2‒ breast cancer and showed that the fusion protein could be targeted using ribavirin. These insights may complement ongoing efforts in precision oncology to extend the clinical benefits of genomics-guided precision treatments.

## Methods

### Specimens and clinical data

All tissue samples utilized in this study were acquired following the approval of the Fudan University Shanghai Cancer Center Ethics Committee, with each patient providing written informed consent. The research was conducted in accordance with recognized ethical guidelines. FUSCC-BRCA is a multiomics cohort comprising 1226 Chinese patients with breast cancer treated at the Department of Breast Surgery at FUSCC between September 2009 and October 2015. This cohort was described in our earlier study^54^. We also included data from the TCGA cohort. The data for the TCGA cohort were downloaded from the TCGA website (https://www.cancer.gov/tcga). The fusion genes in the TCGA cohort were downloaded from the FusionGDB website (https://compbio.uth.edu/FusionGDB2).

### Tissue procurement and RNA extraction

Breast tumor tissues were obtained from the FUSCC tumor bank. Total RNA was extracted from the tissues or cell lines using TRIzol reagent (Invitrogen), according to the manufacturer’s instructions.

### RT-PCR and genomic PCR

Complementary DNA was synthesized with the HiScript III 1st Strand cDNA Synthesis Kit (+gDNA wiper) (Vazyme). To amplify *GAPDH*, *ATF3*, and *ATF4*, RT-PCR was performed with Taq Pro Universal SYBR qPCR Master Mix (Vazyme). For amplification of *KAT6B::ADK* fusions, RT-PCR or genomic PCR was performed with 2× Phanta Max Master Mix (Vazyme). PCR products from the genomic PCR were subsequently purified by Sanger sequencing (Sangon Biotech). The primer sequences and PCR conditions are listed in Supplementary Table S1.

### GSEA and GSVA

GSEA was performed using the GSEA preranked algorithm in the GSEA software (v.3.0) and the Molecular Signature Database hallmark gene sets (v.6.2)^55,56^. The outputs of DESeq2 were used to generate the ranked gene list^57^. Using the “gsva’ function in the R package “gene set variation analysis (GSVA)” (v.1.40.1)^58^, single-sample GSEA scores were determined for each sample.

### Cell lines and cell culture

The human breast cancer cell lines MCF7, T47D, ZR751, and CAMA-1; the human breast cell line MCF10A; and the human embryonic kidney cell line HEK293T were obtained from the American Type Culture Collection (ATCC). Mouse embryo fibroblast NIH-3T3 cells were also purchased from the ATCC. The identify of each cell line was via short tandem repeat profiling. MCF10A cells were cultured as previously described^59^. HEK293T and MCF7 cells were cultured in Dulbecco’s modified Eagle’s medium (DMEM) supplemented with 10% fetal bovine serum (FBS). T47D and ZR751 cells were cultured in Roswell Park Memorial Institute (RPMI) 1640 medium supplemented with 10% FBS. CAMA-1 cells were cultured in Eagle’s minimum essential medium (EMEM) supplemented with 10% FBS. All cell lines were cultured in a humidified incubator at 37 °C and 5% CO_2_. Only cells that were thawed within 6 months were used in the current study. To ensure the maintenance of phenotypes, cell morphology, doubling times, and mycoplasma contamination were recorded regularly.

### Fusion detection and fusion filter

Fusion detection was performed with the STAR-fusion^60^ and Ariiba^11^ pipelines, using GRCh37 as the reference genome. Fusions were called using at least two tools. Fusions were removed if the partners were the same gene; if the genes appeared on the blacklist (including uncharacterized genes, immunoglobulin genes, and mitochondrial genes); if they were paralogs; if the fusion was from a list of normal panel fusions; if one partner was promiscuous with 25 or more partners; or if the partner genes were within 300 kb. In addition, across all samples for a particular fusion pair, we required at least one sample to have two or more junction reads or one sample to have one or more spanning reads.

### Kinase domain analysis

We curated a list of kinase genes from previous studies and public databases^6^. Kinase domain status was determined on the basis of the reported gene fusion breakpoints using Arriba. Afterward, we compared this list with the UniProt/PFAM domain database (http://www.uniprot.org/database) to retain those with an annotated kinase domain.

### Read count of fusions in tumors

We used the Arriba fusion detection tool to calculate the total supporting reads for each fusion event using the following formula: Total Supporting Reads = split reads1 + split reads2 + discordant mates.

### DNA constructs, transfection, and viral transduction

Full-length cDNAs of *KAT6B::ADK*, *ADK::PCDH15*, *ADK::LRMDA*, *LRDMA::ADK*, and Δ*KAT6B* containing full-length open reading frames (ORFs) were amplified from fusion-positive tumors with 2× Phanta Max Master Mix (Vazyme). *ADK* full-length cDNA (NM_001369123.1) was synthesized by Sangon Biotech. Mutants of *KAT6B::ADK* fusion (*KAT6B::ADK*(175)-ΔIDR1, *KAT6B::ADK*(175)-ΔIDR2, *KAT6B::ADK*(175)-ΔIDR3, *KAT6B::ADK*(260)-ΔIDR1, *KAT6B::ADK*(260)-ΔIDR2 and *KAT6B::ADK*(260)-ΔIDR3) were synthesized with SYNBIO TECHNOLOGIES. For *KAT6B::ADK* fusions, Δ*KAT6B* or *ADK* cDNA was subcloned and inserted into a lentiviral vector. The EGFP tag was fused to the C-terminus of these genes and inserted into pCDH-CMV plasmids. The AcGFP1 tag was fused to the N-terminus of these genes and inserted into pLVX-CMV plasmids. After validation by Sanger sequencing (Sangon Biotech), the shRNA oligo sequences for *KAT6B::ADK* fusion were inserted into the pLKO.1-Puro vector. *ATF4* siRNAs (#1: CUCCCAGAAAGUUUAACAATT; #2: CUGCUUACGUUGCCAUGAUTT) were synthesized by Synbio Technologies Co., Ltd. The sequences are listed in Supplementary Table S2. These constructs were infected by lentivirus into cells, and stable cell lines containing the constructs were selected using flow cytometry sorting against the GFP selection marker or screened with 2 μg/mL puromycin for 1 week.

### Immunoblotting analysis and antibodies

For immunoblot analysis, total proteins were extracted by homogenizing the cells in RIPA lysis buffer (50 mM Tris-HCl, pH 7.4, 1% Nonidet P-40, 0.25% sodium deoxycholate, 0.1% sodium dodecyl sulfate, 150 mM NaCl, and 1 mM EDTA) supplemented with protease and phosphatase inhibitors (Selleck, #B14001 and #B15001, respectively). ~20–50 micrograms of protein extracts were denatured in sample buffer, separated by SDS-PAGE, and transferred onto a PVDF membrane (Millipore, #IPVH00010). The membranes were blocked with 10% nonfat milk in 1× TBST (0.9% NaCl, 10 mM Tris-HCl, pH 7.5, containing 0.05% Tween 20) at room temperature for 1 h and then incubated overnight at 4 °C with primary antibodies, followed by incubation with a horseradish peroxidase-conjugated secondary antibody. The signals were then visualized with an enhanced chemiluminescence system (Clarity Western ECL Substrate and Amersham Imager 600; GE Healthcare). The list of antibodies used for immunoblotting is provided in Supplementary Table S3.

### Cell migration assays

For cell migration and invasion assays, MCF7 cells were suspended in serum-free medium and seeded (5 × 10^4^ cells per well) in the upper chambers (Corning). Serum-enriched medium (DMEM containing 20% FBS) was added to the lower chambers of 24-well plates. After 48 h of incubation, the migrated MCF7 cells were stained with 0.5% crystal violet, photographed, and counted with ImageJ (National Institutes of Health, Bethesda, MD, USA). In addition, to exclude the secondary effect of cell proliferation caused by *KAT6B::ADK*, cells were treated with 10 μg/mL mitomycin C for 2 h to inhibit proliferation prior to performing cell migration assays.

### Wound-healing assay

To measure cell migration, the indicated cells were seeded in 6-well plates (2 × 10^6^ cells per well) and incubated at 37 °C. Upon reaching 100% confluence, the cell monolayers were scratched with sterile 20 μL pipette tips and washed with medium to remove any detached cells. Images were acquired at 0 and 24 h (or 36 h), and the rate of wound healing (% coverage area) was calculated and analyzed.

### Cell proliferation and clonogenic assays

Engineered stable MCF7 and NIH-3T3 cells were seeded at a density of 3000 cells/well or 2000 cells/well in a 96-well plate. Cell proliferation was measured with a Cell Counting Kit-8 assay at different time points (Biosharp). Absorbance was measured at 450 nm using a SpectraMax M5 (Molecular Devices). For tamoxifen or ribavirin dose curves, stable MCF7 and T47D cells were seeded at a density of 10,000 cells/well in a 96-well plate and treated with the vehicle or different doses of tamoxifen or ribavirin. Cell proliferation was measured using the Cell Counting Kit-8 assay after 48 h or 60 h of treatment. For the clonogenic assay, stable MCF7 or NIH-3T3 cells were seeded at a density of 1000 cells/well in a 6-well plate in triplicate and cultured for 2 weeks or 7 days. For the clonogenic assay with tamoxifen treatment, stable MCF7 cells were seeded at a density of 10,000 cells/well in 24-well plates. After attachment to the plate, the cells were treated with 0.1% DMSO (vehicle) or tamoxifen at the indicated concentrations for MCF7 cells for 2 days before the chemical was replaced with fresh growth medium. The cells were then fixed with methanol and stained with 1% crystal violet. Clonogenicity was photographed and quantified using ImageJ software.

### 3D culture and indirect immunofluorescence staining of MCF10A acini

3D culture of MCF10A cells was performed as described previously^59^. Briefly, fusion-expressing MCF10A cells were diluted to a final concentration of 25,000 cells/mL, and Matrigel (Corning) was added at a 1:1 ratio. The matrigel–cell mixture was plated in 8-well chamber slides (Lab-tek), with 400 μL in each well. The cells were grown in a 5% CO_2_ humidified incubator. To stain cell–cell junctions, the acini were stained with anti-β-catenin at a 1:100 dilution and secondary Alexa Fluor 488 at a 1:200 dilution. DAPI was used to distinguish the nuclei. Confocal microscopy (Leica TCS SP8 confocal microscopy) was used for image capture. LAS X (Leica) software was used for quantification, and at least 100 acini were analyzed per construct.

### Live cell imaging

Cells were grown on *Φ* 15 mm glass-bottom dishes coated with 0.1% polylysine (NEST, #801002), and images were captured with a Leica TCS SP8 confocal microscopy system with a 63× oil objective (numerical aperture (NA) = 1.2). Cells were imaged on a heated stage (37 °C) and supplemented with warm (37 °C) humidified air. Fluorescent images were processed and assembled into figures using LAS X (Leica) and Fiji software.

### FRAP

The FRAP assay was conducted with the FRAP module of a Leica SP8 confocal microscopy system and a Leica Thunder microscope. The AcGFP1-tagged fusion proteins were bleached with a 488 nm laser beam. Bleaching was focused on a circular or rectangular region of interest (ROI) using 100% laser power, and time-lapse images were collected. The fluorescence intensity was measured using Fiji. The background intensity was subtracted, and the values were reported relative to the prebleaching time points. R was used to plot and analyze the FRAP results.

### Animal studies

The animal experiments were conducted at Shanghai Laboratory Animal Center, Chinese Academy of Sciences, under specific pathogen-free conditions at 22 °C ± 2 °C and 50%–60% humidity, with a 12 h light cycle and food and water ad libitum. All animal experiments were approved by the Institutional Animal Care and Use Committee of FUSCC (FUSCC-IACUC-2024225). The maximum tumor size/burden was not exceeded in any of the experiments.

Six-week-old NOD/SCID female mice were used in the experiments shown in Fig. 4. Six-week-old female BALB/c-nude mice were used in the experiments shown in Supplementary Fig. S7. To allow MCF7 xenograft growth, on the day before cell injection, a 17β-estradiol-releasing pellet (Innovrsrch) was inserted into the intrascapular subcutaneous region. 8 × 10^5^ NIH-3T3 cells were injected subcutaneously into one flank of each mouse. Alternatively, 3 × 10^6^ MCF7 cells were orthotopically injected into the fourth abdominal fat pad. Treatment was initiated when the tumors appeared as established palpable masses (~3 weeks after cell injection). In each experiment, mice were randomly assigned to one of the following groups: control (treated with DMSO) or tamoxifen (45 mg/kg/d in CMC-NA, oral gavage). The tumor volume was measured using a vernier caliper and was calculated using the equation *V* = (*L* × *W*^2^)/2, where *L* refers to the longer diameter and *W* indicates the shorter diameter perpendicular to *L*. On the indicated days, the mice were euthanized, and the tumors were photographed, collected, and fixed.

### Chemicals

ABT-702 dihydrochloride (MCE, Cat# HY-103161), tamoxifen (Selleck, Cat# S1238), ribavirin (Selleck, Cat# S2504), CMC-Na (Selleck, Cat# S6703), adenosine dialdehyde (MCE, Cat# HY-123055), GCN2-IN-1 (MCE, Cat# HY-100877), cycloheximide (MCE, Cat# HY-12320), and D-luciferin sodium (MCE, Cat# HY-12591) were used.

### ATP level measurement

ATP within the cell suspension and supernatants was quantified using a luminescence-based assay (A22066, Invitrogen) in strict adherence to the guidelines.

### Bioluminescence Imaging

Bioluminescence was detected using a PerkinElmer IVIS Imaging System in accordance with the manufacturer’s recommendations and protocols. For metastasis detection, 10 min prior to sacrifice, the mice were injected with 150 mg/kg d-luciferin potassium (HY-12591B, MCE), and the resected organs were immediately imaged and analyzed using Living Image software (PerkinElmer, Inc.).

### PDOs

Human organoids were obtained from surgical specimens of patients who underwent surgery at the Department of Breast Cancer, FUSCC. All clinical samples were obtained with informed consent from each patient, who signed an informed consent form approved by the FUSCC Ethics Committee (Protocol number: 050432-4-1911D). PDOs were cultured following a previously published protocol^61^. For organoid drug treatment, organoids in good condition were diluted to 40 μL/organoid, plated onto 384-well plates (781976-SIN, Greiner), and cultured for another 3 days before being subjected to drug treatments. Organoid cell viability was evaluated using the CellTiter-Glo 3D Cell Viability Assay (G9683, Promega) after treatment with the indicated drugs for 5 days.

### Organoid lentiviral transduction

Organoids were cultured and dissociated into single cells, passed through a 40-µm filter, and resuspended in medium supplemented with polybrene (8 mg/mL). Cells were spin-infected (700× *g*, 90 min, 25 °C) on low-adhesion plates (Corning), followed by rotation incubation at 37 °C for 4–5 h. Subsequently, the organoids were centrifuged at 400× *g* for 3 min at 4 °C and seeded in Matrigel. Selection began 3–5 days posttransduction with puromycin (2 µg/mL, Gibco).

### RNA sequencing

RNA was isolated from MCF7 cells with TRIzol reagent (Invitrogen). RNA samples were then subjected to library construction for RNA sequencing using the VAHTS mRNA-seq V2 Library Prep Kit for Illumina (Vazyme, NR601-01). The resulting libraries were subsequently using a HiSeq-2500 platform (Illumina). The obtained sequencing reads were aligned with the hg38 genome assembly using the HISAT2 software. Cufflinks were used to calculate the transcripts per kilobase of exon model per million mapped reads for each gene.

### Statistical analysis

Statistical analyses were conducted with GraphPad Prism, version 9.3. The Kaplan–Meier method was used to generate survival curves, which were subsequently compared using log-rank tests. The RFS was calculated from the date of surgery until the identification of recurrence. OS was calculated from the date of surgery to either the date of death or the last follow-up. DMFS was defined as the period between surgery and metastasis or the end of follow-up. Patients who did not experience any events were censored on the date of the last follow-up. Statistical significance was set at *P* < 0.05. The statistical methods employed are specified in the corresponding figure legends. All experiments were repeated three times, and the results from the representative experiments are shown.

## Supplementary information

Supplementary information

## Data Availability

The sequencing data have been deposited in The National Omics Data Encyclopedia (NODE) (accession numbers OEP003358, OEP003049, OEP000155, OEP001027, OEP003469, and OEP004654). The RNA sequencing data for our study have been deposited into the Genome Sequence Archive (GSA) database under accession code PRJCA036608 (https://bigd.big.ac.cn/gsa-human/browse/HRA015446). All other data are provided in the article or Supplementary Materials.
