# Supplementary material for: Landscape of gene fusions in hormone receptor-positive breast cancer reveals ADK fusions as drivers of progression and potential therapeutic targets
Source: Cell Discov. 2025 Nov 11;11:89. doi: 10.1038/s41421-025-00830-z (PMC12603066; doi:10.1038/s41421-025-00830-z)
Supplement: Supplementary file 1 — Supplementary information [file 41421_2025_830_MOESM1_ESM.pdf]

Supplementary Materials for

Landscape of gene fusions in hormone receptor-positive breast cancer revealed *ADK* fusions as drivers of progression and potential therapeutic targets

Yang Ou-Yang et al.

*Corresponding author. Email: yizhoujiang@fudan.edu.cn (YJ), zhimin_shao@yeah.net (ZS), 15111230037@fudan.edu.cn (XJ).

This file includes:

Supplementary Figs. S1 to S12

Supplementary Tables S1 to S3

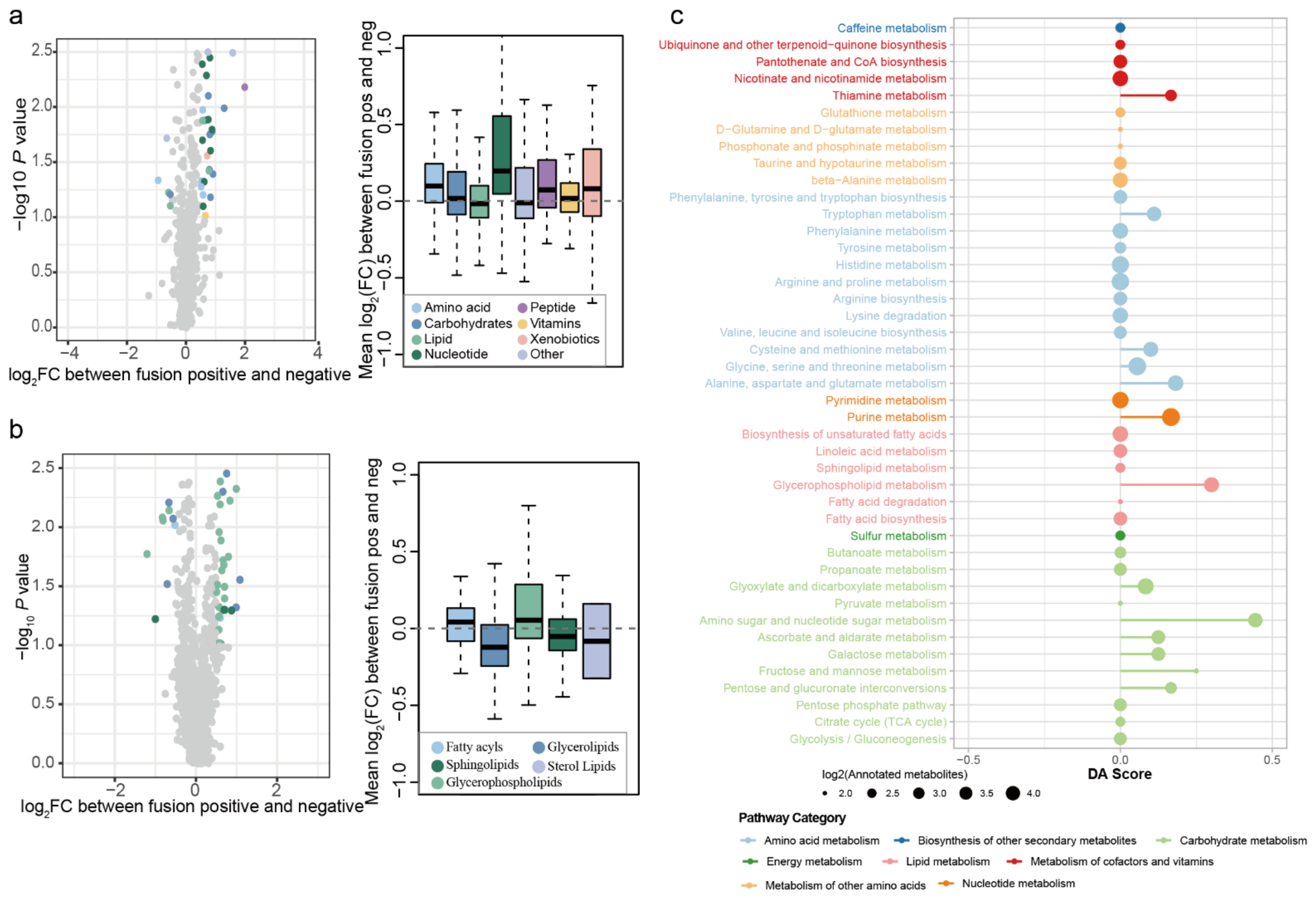

Supplementary Fig. S1 Association of fusion genes with metabolic dysregulation. a A volcano plot of the 669 annotated polar metabolites. Differentially abundant metabolites of different categories were individually color coded. Log2 fold change (FC) of different categories of polar metabolites between the fusion genes positive and negative tumors. The dashed line represents the same level of metabolite abundance between the fusion genes positive and negative tumors. *P* values are calculated using the two-sided Kruskal–Wallis test and adjusted by the Benjamini–Hochberg procedure. **b** A volcano plot of the 1312 annotated lipids metabolites. Differentially abundant metabolites of different categories were individually color coded. Log2 FC of different categories of lipids metabolites between the fusion genes positive and negative tumors. The dashed line represents the same level of metabolite abundance between the fusion genes positive and negative tumors. *P* values are calculated using the two-sided Kruskal–Wallis test and adjusted by the Benjamini–Hochberg procedure. **c** A pathway-based analysis of metabolomic changes between the fusion positive and negative tumors. The differential abundance (DA) score captures the average, gross changes for all metabolites in a pathway. A score of 1 indicates that all measured metabolites in the pathway increase in the tumor compared with fusion genes negative tissues, and a score of −1 indicates that all measured metabolites in a pathway decrease. Pathways with no less than three

measured metabolites were used for DA score calculation. *P* values are calculated using the two-sided Kruskal–Wallis test and adjusted by the Benjamini–Hochberg procedure.

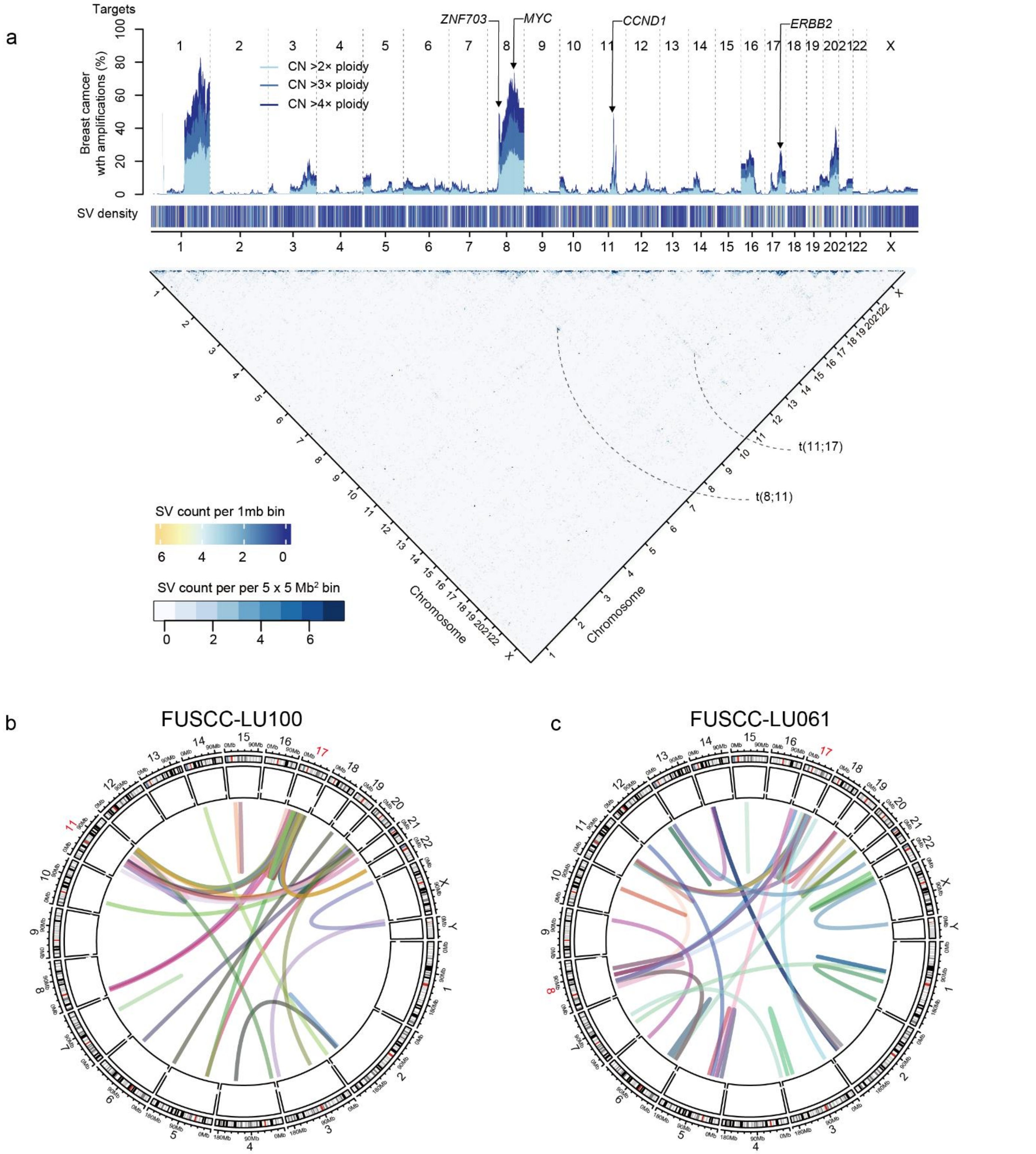

Supplementary Fig. S2 Interchromosomal translocations are frequently associated with focal amplifications in HR+/HER2– breast cancer. a Copy number profile and structural variations (SVs) in 573 HR+/HER2– breast cancers. The fraction of tumors containing amplified genomic regions with different copy number thresholds (top) and frequencies of SVs connecting two genomic regions (bottom) are shown. CN, copy number. **b, c** Circo plots show copy numbers and the SVs in two cases of HR+/HER2– breast cancer.

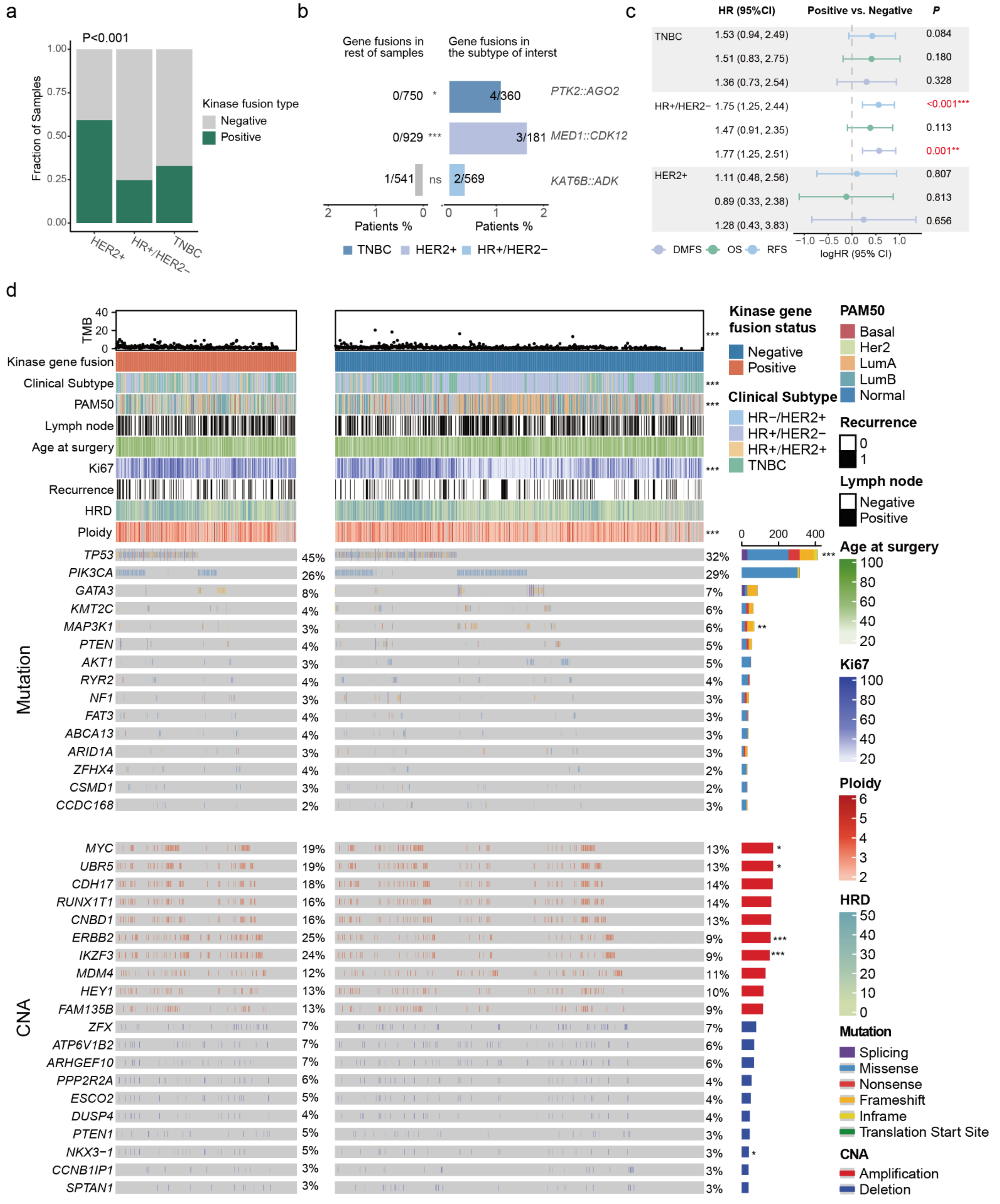

Supplementary Fig. S3 Clinicopathological and molecular landscape of kinase fusion-positive breast cancer. a The data shows the proportions of breast cancer IHC subtypes with or without kinase fusion genes. **b** Comparison of the prevalence of gene fusions in different breast cancer IHC subtypes. The number of patients with each fusion and the total number of patients with

each breast cancer IHC subtype are shown on each bar. *P* values were tested using Fisher's exact test. ***, $P < 0.001$, *, $P < 0.05$, ns, $P \geq 0.05$. **c** Prognostic significance of fusion genes in different breast cancer IHC subtypes. **d** Breast cancer samples with mutation and/or copy number alteration data are ordered by fusion gene subtype and mutation profile, with clinical and molecular features annotation. Asterisks indicate associations with breast cancer clinical subtypes, PAM50 subtypes, lymph node status, recurrence status and somatic copy number alterations were tested with Pearson's chi-square test; TMB, age, Ki67, ploidy and HRD score were tested with the Kruskal–Wallis test; somatic mutations were tested using Fisher's exact test. ***$P < 0.001$, **$P < 0.01$, *$P < 0.05$.

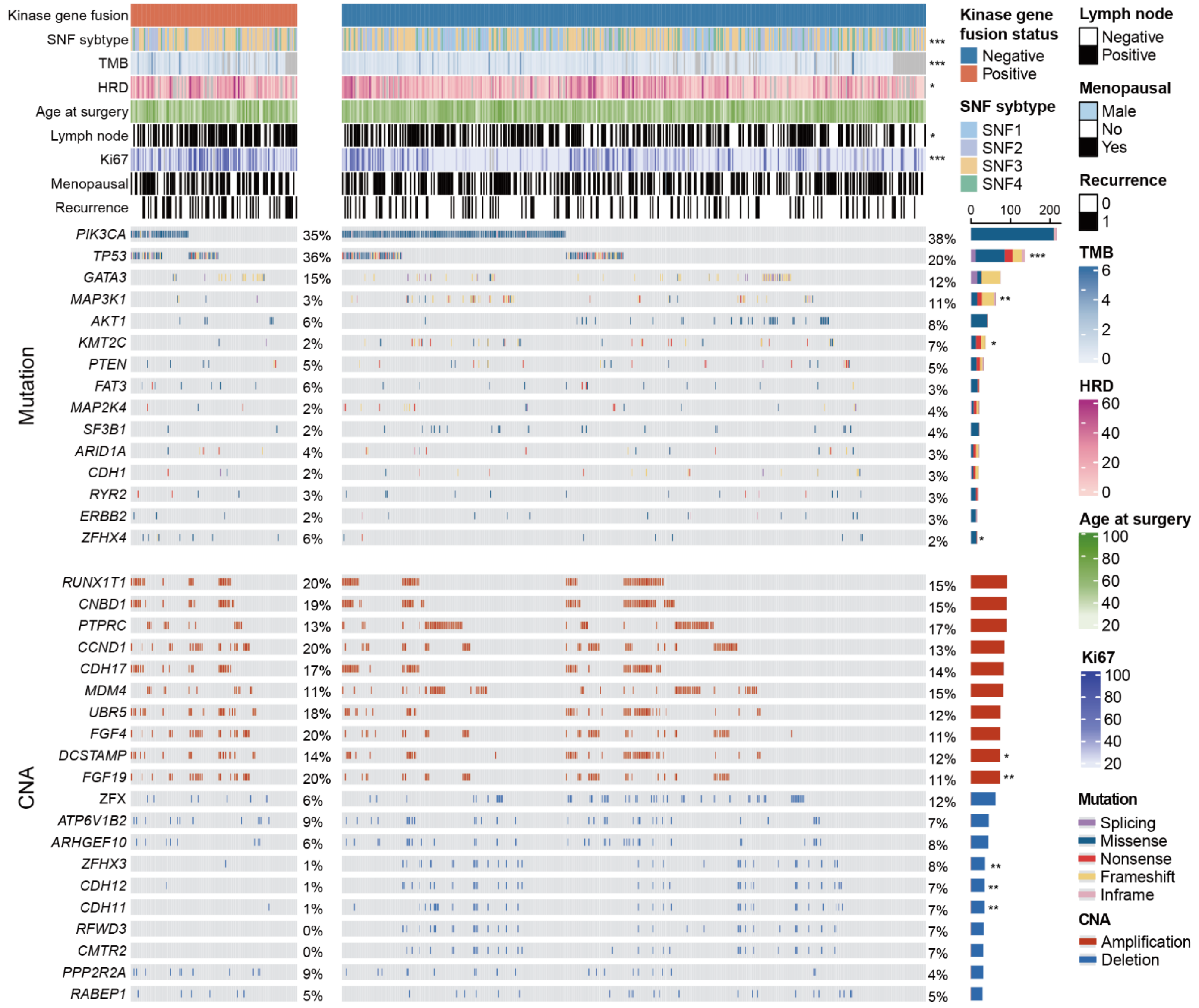

Supplementary Fig. S4 Distinct molecular characteristics of kinase fusion-positive HR+/HER2– breast cancer. HR+/HER2– breast cancer samples with mutation and/or copy number alteration data are ordered by fusion gene subtype and mutation profile, with clinical and molecular features annotation. Asterisks indicate associations with HR+/HER2– breast cancer similarity network fusion (SNF) subtypes, lymph node status, menopausal status, recurrence status and somatic copy number alterations were tested using Pearson's chi-square test; TMB, age, Ki67 and HRD score were tested using the Kruskal–Wallis test; somatic mutations were tested using Fisher's exact test. ***$P < 0.001$, **$P < 0.01$, *$P < 0.05$.

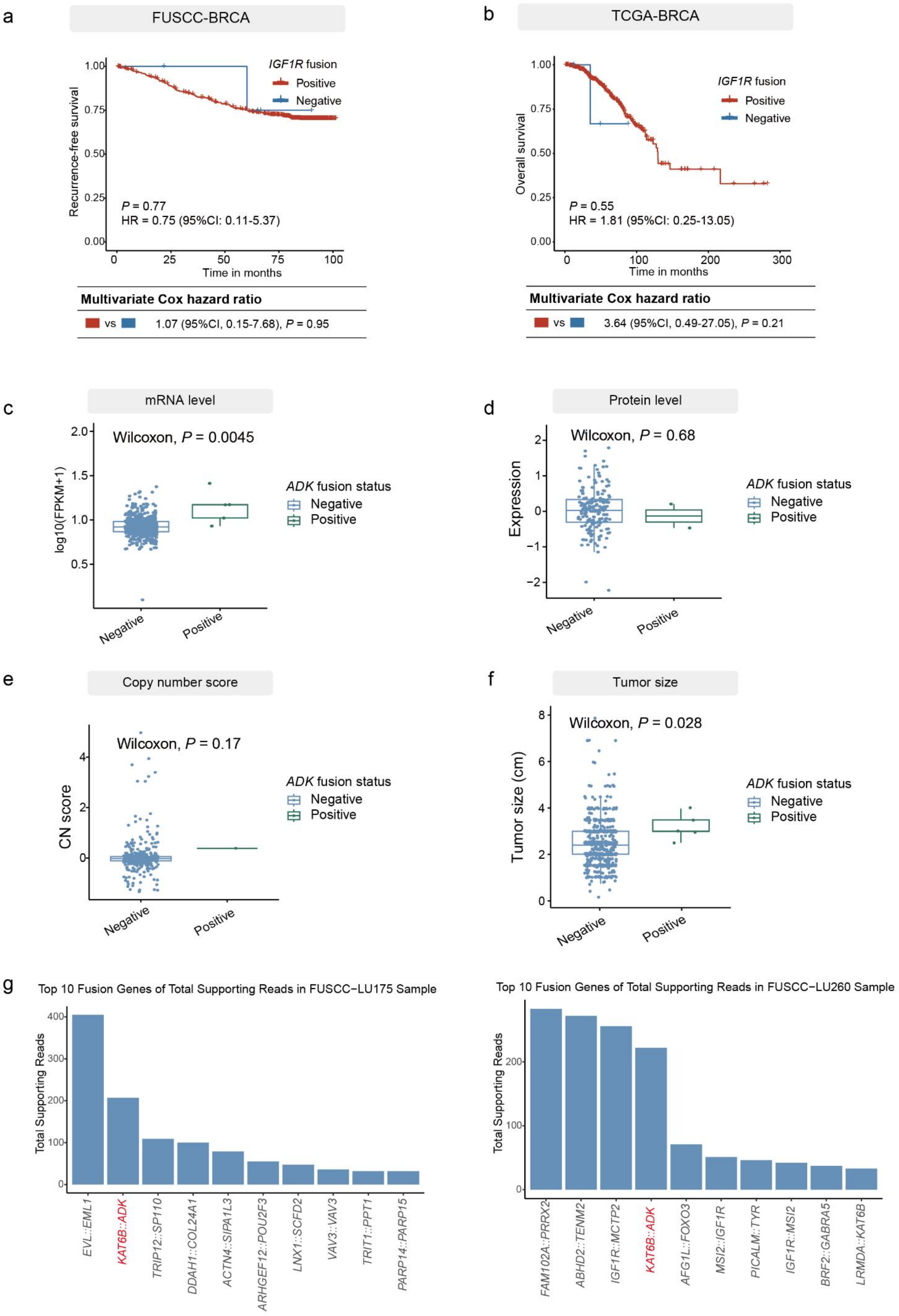

Supplementary Fig. S5 Characteristics of kinase fusion genes. a, b Kaplan–Meier analysis of RFS in patients with or without *IGF1R* fusion genes in FUSCC-BRCA (**a**) and TCGA-BRCA (**b**)

cohort respectively. *P* values calculated by Mantel–Cox log-rank test. A Multivariate Cox proportional hazards model was used to obtain hazard ratios and *P* values, adjusting for the confounders of age and T stage. **c–f** Distribution of the mRNA expression level (**c**), protein expression level (**d**), copy number scores (**e**), tumor size (**f**) across the *ADK* fusion gene subtype in HR+/HER2– breast cancer. **g** Top 10 fusion events by total supporting reads in FUSCC-LU175 (left) and FUSCC-LU260 (right) tumor sample are shown.

.

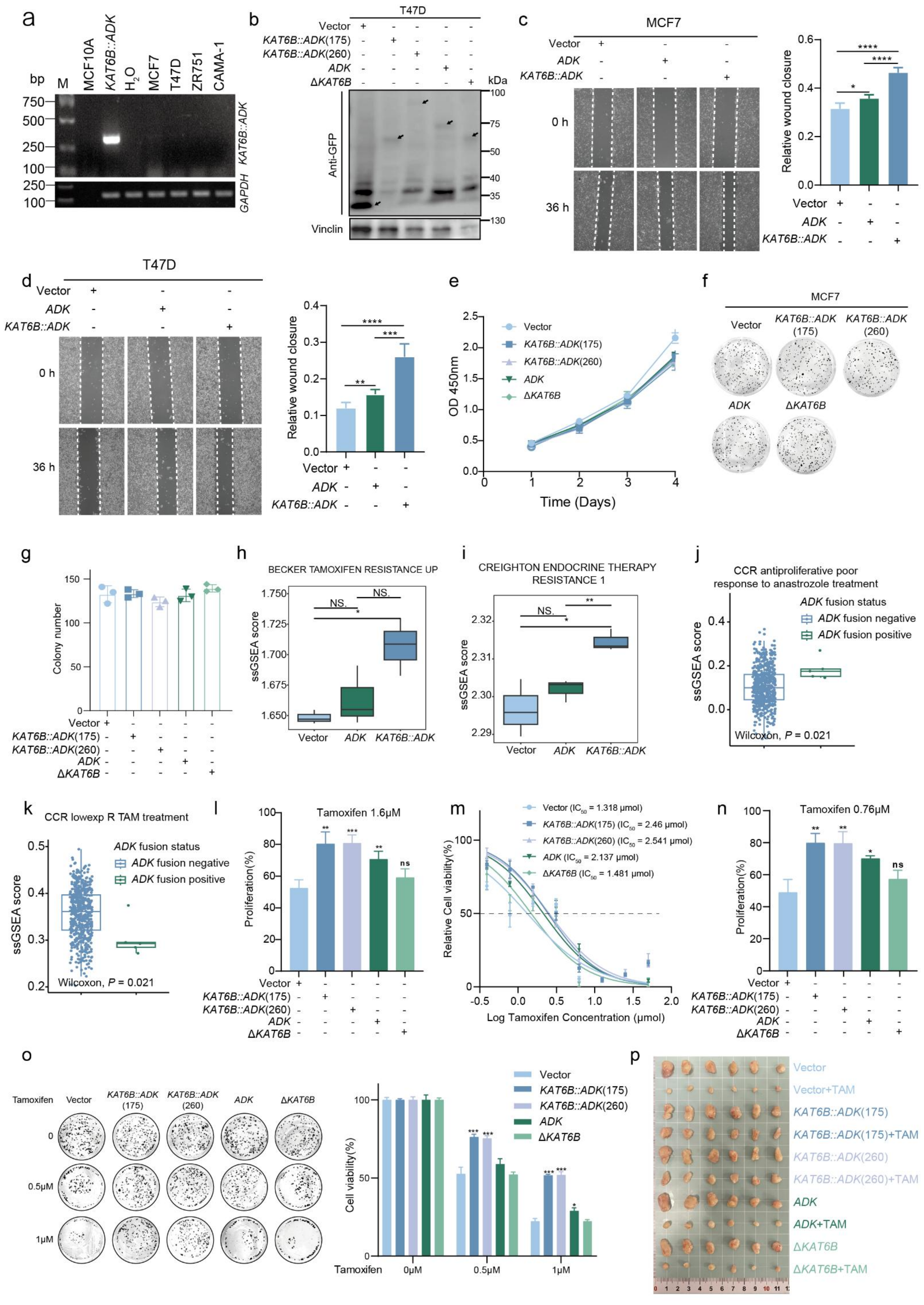
a
bp
M
MCF10A
KAT6B::ADK
H_2O
MCF7
T47D
ZR751
CAMA-1
750
500
250
100
KAT6B::ADK
GAPDH
b
T47D
Vector
KAT6B::ADK(175)
KAT6B::ADK(260)
ADK
ΔKAT6B
kDa
Anti-GFP
Vinclin
c
MCF7
Vector
ADK
KAT6B::ADK
0 h
36 h
Relative wound closure
d
T47D
Vector
ADK
KAT6B::ADK
0 h
36 h
Relative wound closure
e
OD 450nm
Time (Days)
Vector
KAT6B::ADK(175)
KAT6B::ADK(260)
ADK
ΔKAT6B
f
MCF7
Vector
KAT6B::ADK (175)
KAT6B::ADK (260)
ADK
ΔKAT6B
g
Colony number
h
BECKER TAMOXIFEN RESISTANCE UP
ssGSEA score
NS.
Vector
ADK
KAT6B::ADK
i
CREIGHTON ENDOCRINE THERAPY RESISTANCE 1
ssGSEA score
j
CCR antiproliferative poor response to anastrozole treatment
ADK fusion status
ADK fusion negative
ADK fusion positive
ssGSEA score
Wilcoxon, P = 0.021
k
CCR lowexp R TAM treatment
ADK fusion status
ADK fusion negative
ADK fusion positive
ssGSEA score
Wilcoxon, P = 0.021
l
Tamoxifen 1.6μM
Proliferation(%)
m
Vector (IC50 = 1.318 μmol)
KAT6B::ADK(175) (IC50 = 2.46 μmol)
KAT6B::ADK(260) (IC50 = 2.541 μmol)
ADK (IC50 = 2.137 μmol)
ΔKAT6B (IC50 = 1.481 μmol)
Relative Cell viability(%)
Log Tamoxifen Concentration (μmol)
n
Tamoxifen 0.76μM
Proliferation(%)
o
Tamoxifen
Vector
KAT6B::ADK (175)
KAT6B::ADK (260)
ADK
ΔKAT6B
0
0.5μM
1μM
Cell viability(%)
0μM
p
Vector
Vector+TAM
KAT6B::ADK(175)
KAT6B::ADK(175)+TAM
KAT6B::ADK(260)
KAT6B::ADK(260)+TAM
ADK
ADK+TAM
ΔKAT6B
ΔKAT6B+TAM

Supplementary Fig. S6 Biological characteristics of *ADK* fusion genes. a RT-PCR analyses of *KAT6B::ADK* fusions in HR+/HER2– cells are shown. MCF10A cells were used as a negative control. cDNA isolated from breast cancer samples expressing the *KAT6B::ADK* fusions served as a positive control. *GAPDH* transcripts serve as internal loading control. M, DNA ladder marker. **b** The *KAT6B::ADK*(175), *KAT6B::ADK*(260), *ADK*, and Δ*KAT6B* were overexpressed in T47D cells and validated by immunoblotting for GFP. **c, d** Wound healing assays were performed to evaluate the effects of vector, *KAT6B::ADK* and *ADK* on the migration of the indicated cells. **e–g** CCK-8 assays (**e**) and colony formation assays (**f, g**) were performed to assess the effects of vector, *KAT6B::ADK*, *ADK* and Δ*KAT6B* on the proliferation of MCF7 cells. **h–k** The comparison of signature across groups is shown. Two-sided *P* values were calculated with the Wilcoxon test. **l** MCF7 cells were treated 48h with 1.5 μM tamoxifen and viability was measured using CCK-8 assay. The data are presented as mean ± SD from three independent experiments. **m, n** Dose−response curves with 48 h of tamoxifen treatment in T47D cells. Cell viability normalized to vehicle (DMSO)-treated cells. **o** MCF7 cells stably expressing the indicated constructs were treated with increasing concentrations of tamoxifen for 10 days, followed by colony formation assays. **p** Representative images of tumor tissues are shown.

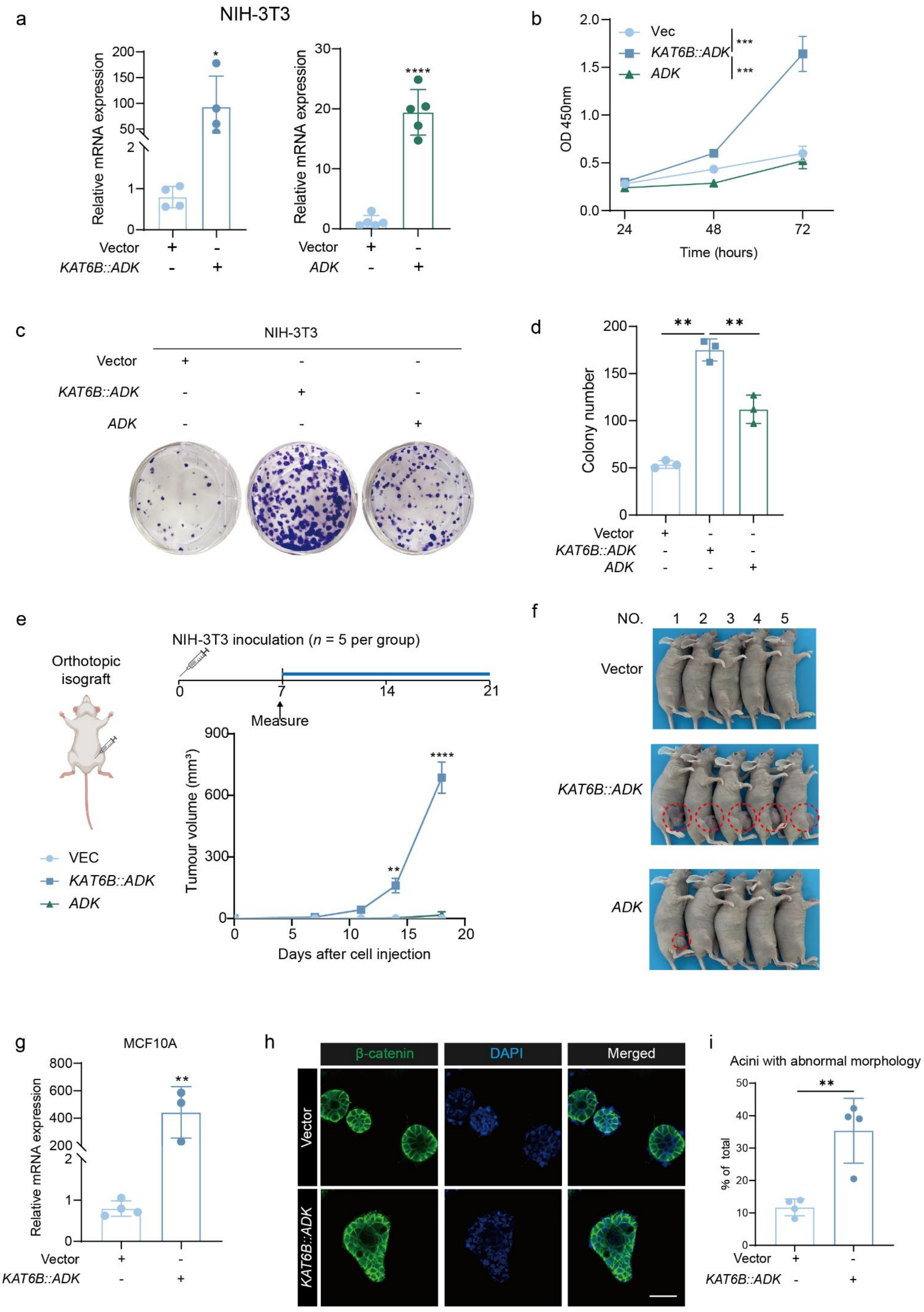
a
NIH-3T3
Relative mRNA expression
Vector
KAT6B::ADK
ADK
b
OD 450nm
Vec
KAT6B::ADK
ADK
Time (hours)
c
NIH-3T3
Vector
KAT6B::ADK
ADK
d
Colony number
e
Orthotopic isograft
NIH-3T3 inoculation (n = 5 per group)
Measure
Tumour volume (mm³)
Days after cell injection
VEC
KAT6B::ADK
ADK
f
NO. 1 2 3 4 5
Vector
KAT6B::ADK
ADK
g
MCF10A
Relative mRNA expression
h
β-catenin
DAPI
Merged
Vector
KAT6B::ADK
i
Acini with abnormal morphology
% of total

Supplementary Fig. S7 Transforming activity of the *KAT6B::ADK* fusion. a The effect of overexpression of *KAT6B::ADK* or *ADK* mRNA level in NIH-3T3 cells is shown. **b–d** CCK-8 assays (**b**) and colony formation assays (**c, d**) were performed to assess the effects of vector, *KAT6B::ADK* and *ADK* on the proliferation of NIH-3T3 cells. **e, f** Growth curve of tumors (**e**) formed following subcutaneous injection of NIH-3T3 cells into the bilateral flanks of BALB/c-nude mice. A representative image (**f**) is shown. Error bars indicate mean ± SEM. **g** The effect of overexpression of *KAT6B::ADK* mRNA level in NIH-3T3 cells is shown. **h, i** MCF10A cells transduced with vector or *KAT6B::ADK* were plated at clonal density in 3D basement membrane culture (**h**). Scale bar, 50 μm. The right graphs show quantitation of deregulated growth and polarization (morphology) (**i**). *P* values are determined by two-tailed Student's *t*-test. ***P* < 0.01.

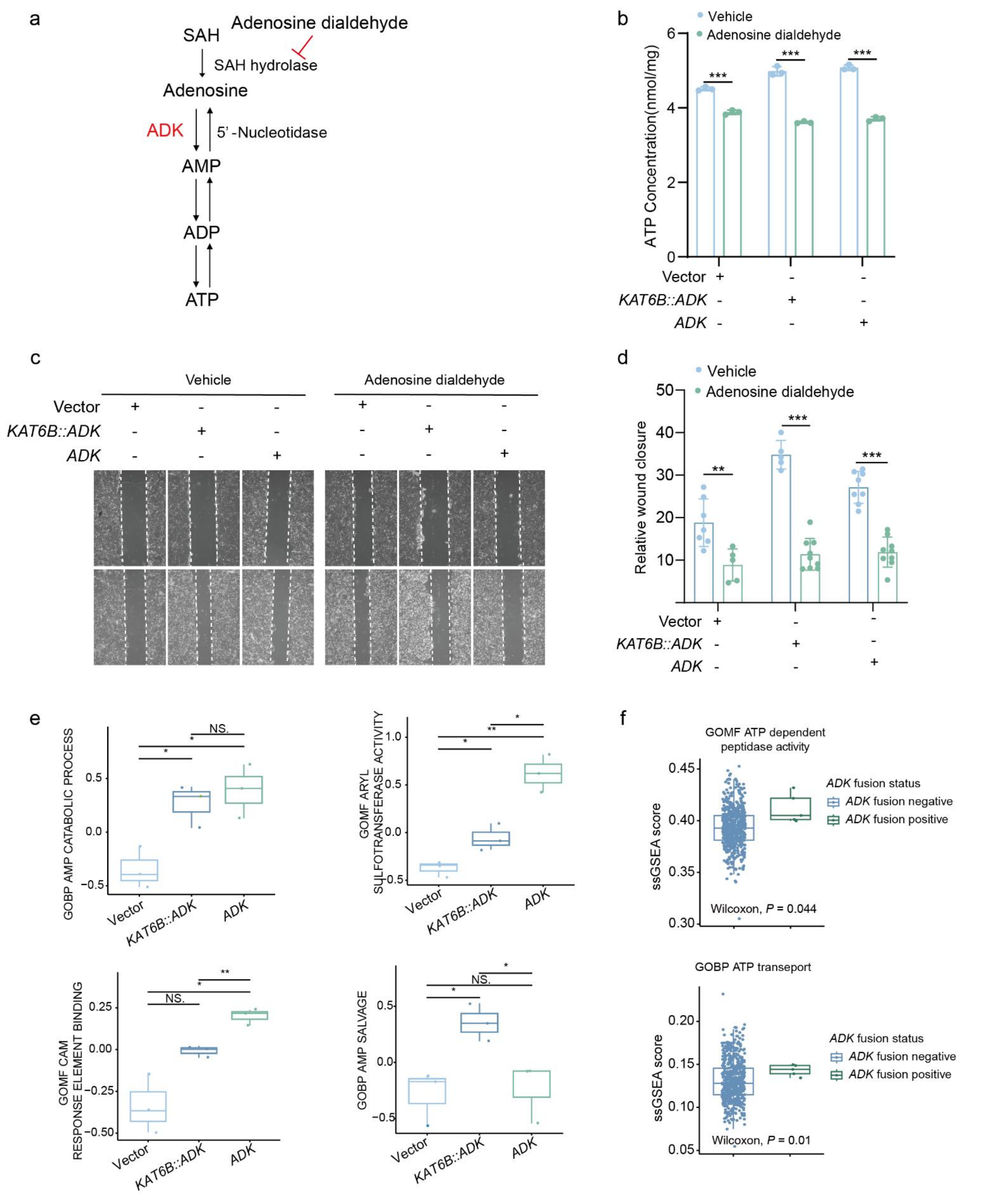

Supplementary Fig. S8 *ADK* fusions activate adenosine metabolism-related pathways. a Schematic representation of adenosine metabolism pathways is shown and highlight the inhibitory site of adenosine dialdehyde. **b** The effects of *KAT6B::ADK* and *ADK* overexpression with adenosine dialdehyde treatment on the production and release of ATP in MCF7 cell are shown. **c, d** Wound healing assays were performed to evaluate the effects of adenosine dialdehyde treatment on the

migration of the indicated cells. Representative images (**c**) and quantification of wound closure (**d**) are shown. **e, f** The comparisons of signature among groups are shown. Two-sided *P* values were calculated with the Wilcoxon test.

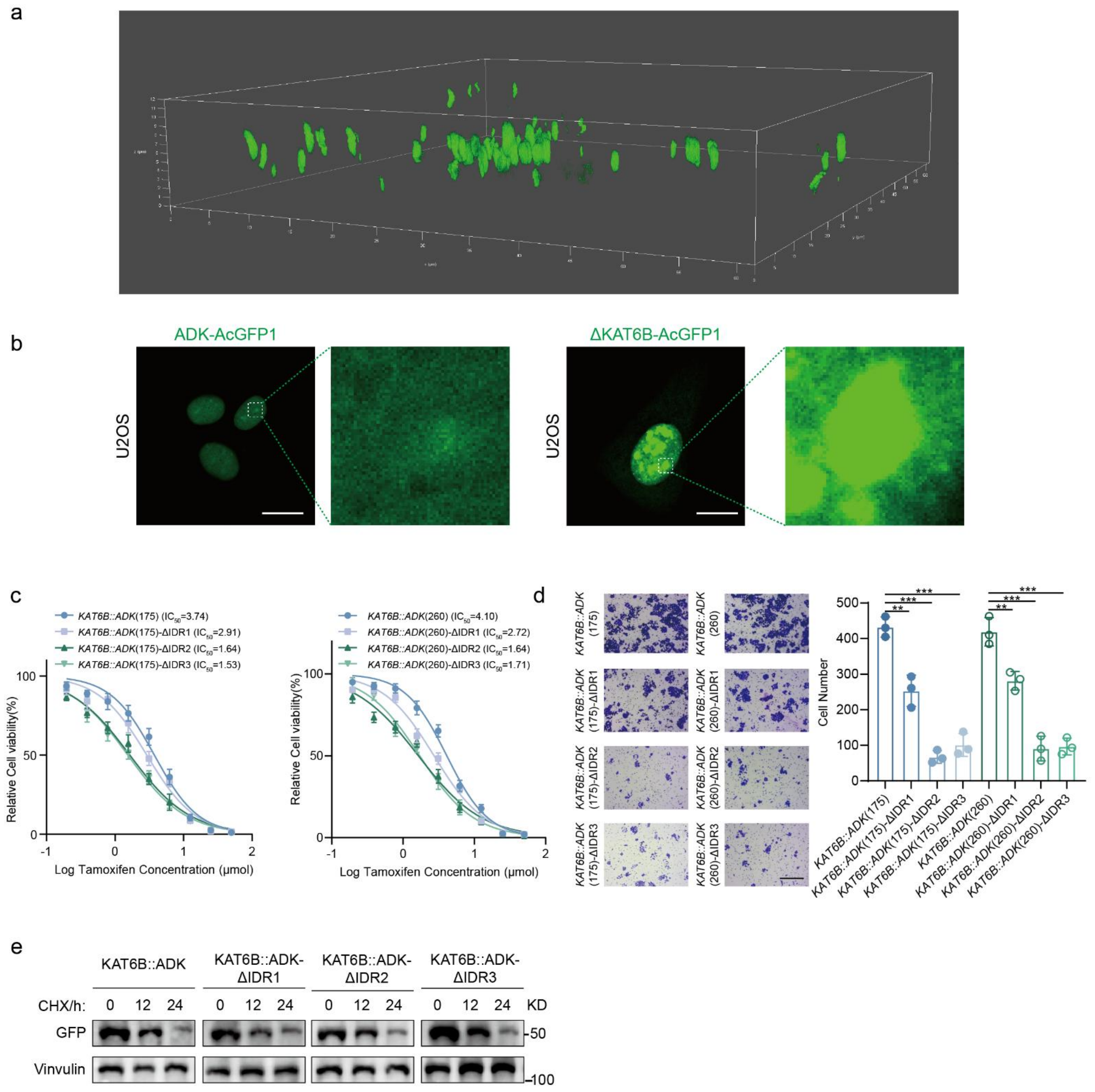

Supplementary Fig. S9 KAT6B::ADK forms phase-separated condensates and modulates cellular functions in breast cancer cells. a A reconstructed 3D image of U2OS cells stably expressing KAT6B::ADK-AcGFP1 is shown. **b** Representative images of U2OS cells stably expressing ADK and ΔKAT6B fusions tagged AcGFP1 proteins are shown. Scale bar: 10 μm. **c** MCF7 cells with overexpression of indicated mutants were treated with increasing concentrations of tamoxifen. The viability was measured with CCK-8 assay. **d** Transwell assays were performed to evaluate the effects of indicated mutants on the migration of MCF7 cells. Representative images and quantification of relative migrated cells are shown. Scale bars, 100 μm. Significance was

determined by Student's *t*-test (***P* < 0.01, ****P* < 0.001). **e** Western blot analyses the protein levels of KAT6B::ADK variants following translational inhibition with cycloheximide (CHX) treatment.

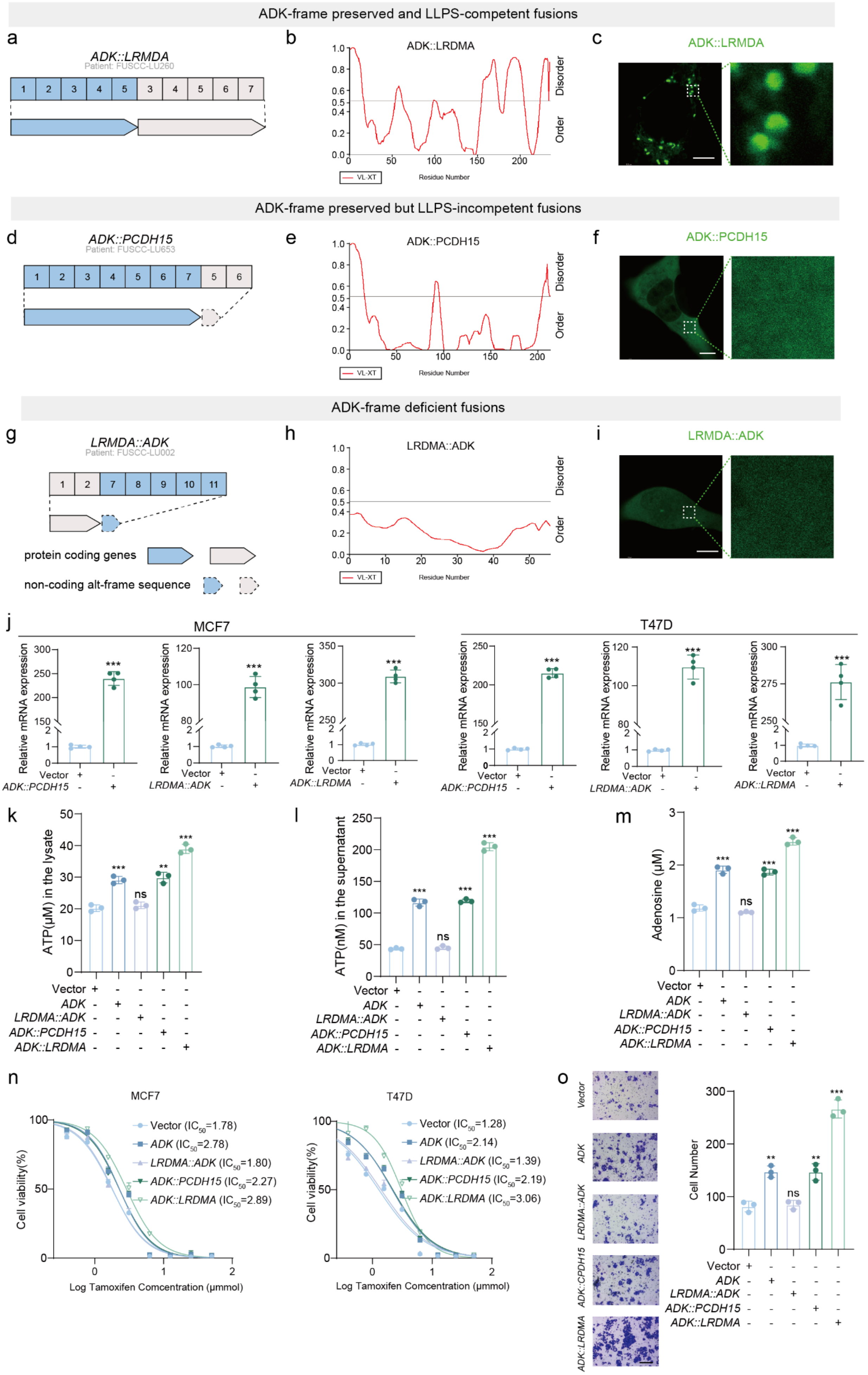
ADK-frame preserved and LLPS-competent fusions
a
ADK::LRMDA
Patient: FUSCC-LU260
b
ADK::LRDMA
Disorder
Order
VL-XT
Residue Number
c
ADK::LRMDA
ADK-frame preserved but LLPS-incompetent fusions
d
ADK::PCDH15
Patient: FUSCC-LU653
e
ADK::PCDH15
f
ADK::PCDH15
ADK-frame deficient fusions
g
LRMDA::ADK
Patient: FUSCC-LU002
h
LRDMA::ADK
i
LRMDA::ADK
protein coding genes
non-coding alt-frame sequence
j
MCF7
T47D
Relative mRNA expression
Vector
ADK::PCDH15
LRDMA::ADK
ADK::LRDMA
k
ATP(μM) in the lysate
l
ATP(nM) in the supernatant
m
Adenosine (μM)
Vector
ADK
LRDMA::ADK
ADK::PCDH15
ADK::LRDMA
n
MCF7
Vector (IC50=1.78)
ADK (IC50=2.78)
LRDMA::ADK (IC50=1.80)
ADK::PCDH15 (IC50=2.27)
ADK::LRDMA (IC50=2.89)
T47D
Vector (IC50=1.28)
ADK (IC50=2.14)
LRDMA::ADK (IC50=1.39)
ADK::PCDH15 (IC50=2.19)
ADK::LRDMA (IC50=3.06)
Cell viability(%)
Log Tamoxifen Comcentration (μmmol)
o
Vector
ADK
LRDMA::ADK
ADK::CPDH15
ADK::LRDMA
Cell Number
Vector
ADK
LRDMA::ADK
ADK::PCDH15
ADK::LRDMA

Supplementary Fig. S10 Structural and functional characterization of *ADK* fusion variants. a, d, g Exon composition of *ADK* fusions genes and evolving proteins identified in FUSCC HR+/HER2– breast tumor samples. **b, e, h** ADK fusions intrinsically disordered regions were predicted by PONDR. **c, f, i** Representative live-cell images of U2OS cells stably expressing fusion proteins tagged with AcGFP1 are shown. Scale bar: 10 μm. **j** Quantitative real-time PCR was used to determine the mRNA levels of *ADK::PCDH15*, *LRDMA::ADK*, and *ADK::LRDMA* in cells overexpressing each construct. Data are presented as mean ± SD. Significance was determined by Student's *t*-test (***$P < 0.001$). **k, l** The effects of *ADK* fusions on the production and release of ATP in MCF7 cell. Significance was determined by Student's *t*-test (**$P < 0.01$, ***$P < 0.001$). **m** ELISA analysis of intracellular adenosine from MCF7 cells transfected with *ADK* fusions. Significance was determined by Student's *t*-test (***$P < 0.001$) **n** MCF7 and T47D cells were treated 48 h with increasing concentrations of tamoxifen and viability was measured with CCK-8 assay. Data are presented as mean ± SD from three independent experiments. **o** Transwell assays were performed to evaluate the effects of vector, *ADK*, *LRDMA::ADK*, *ADK::PCDH15*, and *ADK::LRDMA* on the migration of the MCF7 cells. Representative images and quantification of relative migrated cells are shown. Scale bars, 100 μm. Significance was determined by Student's *t*-test (**$P < 0.01$, ***$P < 0.001$).

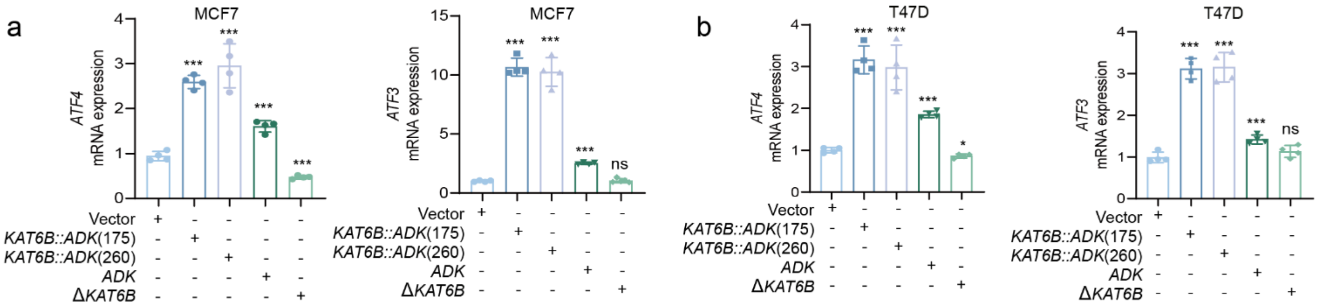

Supplementary Fig. S11 *KAT6B::ADK* fusion induces upregulation of ISR transcription factors *ATF4* and *ATF3*. a, b The *ATF4* and *ATF3* mRNA levels in MCF7 (**a**) and T47D (**b**) cells transfected with vector, *KAT6B::ADK*, *ADK*, and Δ*KAT6B* are shown. (*$P < 0.05$, **$P < 0.01$, ***$P < 0.001$).

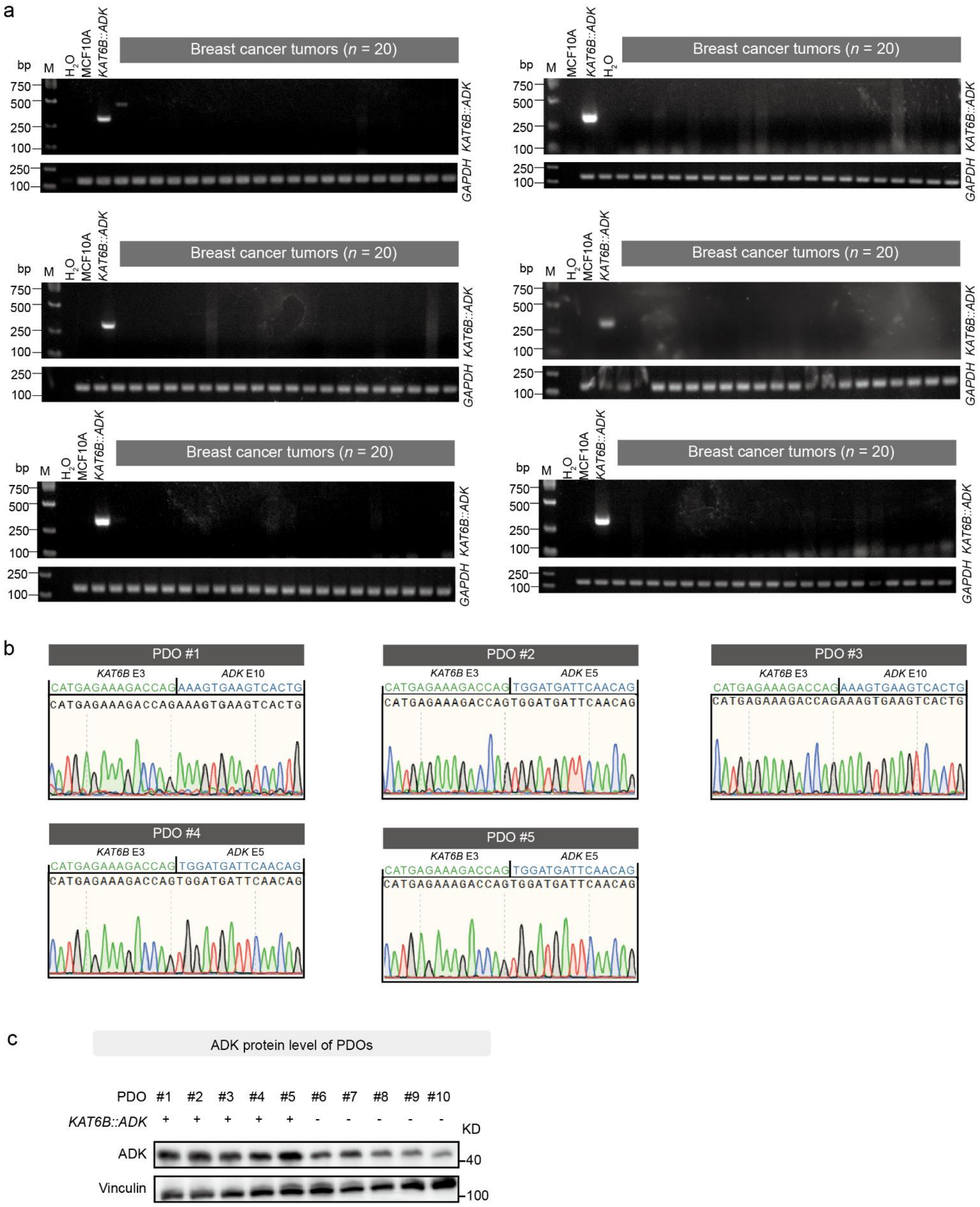

Supplementary Fig. S12 Detection of *KAT6B::ADK* fusion transcripts and ADK protein level in FUSCC-PDOs cohort. a RT-PCR analysis of *KAT6B::ADK* fusion in HR+/HER2– tumors from the FUSCC-PDOs cohort. MCF10A cells were used as a negative control. cDNA derived from breast cancer samples expressing the *KAT6B::ADK* fusion was used as a positive control. *GAPDH* transcripts were used as an internal loading control. M: DNA ladder marker. **b** Chromatograms show

the junction sequences of *KAT6B::ADK* fusion variants detected in PDOs with *KAT6B::ADK* fusions.

c Levels of ADK protein levels in PDOs with or without *KAT6B::ADK* fusions.

Supplementary Table S1. List of oligonucleotides used in this study

Oligonucleotide	Sequence
RT-qPCR primers for human *GAPDH*	F: GCACCGTCAAGGCTGAGAAC<br>R: TGGTGAAGACGCCAGTGGA
RT-qPCR primers for human *ATF3*	F: TTTGCTAACCTGACGCCCTT<br>R: TGACTGATTCCAGCGCAGAG
RT-qPCR primers for human *ATF4*	F: CTCCGGGACAGATTGGATGTT<br>R: GGCTGCTTATTAGTCTCCTGGAC
RT-qPCR primers for human *ADK*	F: AGAGAGCAAGGCTTTGAGACTA<br>R: CCCTTGGGTGAAGATCACGA
RT-qPCR primers for human *KAT6B::ADK* (175)	F: GACCGCAGTACAGGGTCAAT<br>R: CTGGCTTCTCAGGAAAGGTG
RT-qPCR primers for human *KAT6B::ADK* (260)	F: CGCTGTGAATAATGGGAGGT<br>R: CCTGTTGTCACCAGTGATGC

Supplementary Table S2. The sequences of the shRNAs, siRNAs and primers used in this study

Name	Sequence
si*ATF4*	CUCCCAGAAAGUUUAACAATT
sh*KAT6B::ADK* #1	TGAGAAAGACCAGAAAGTGAA
sh*KAT6B::ADK* #2	GAGAAAGACCAGAAAGTGAAG
sh*KAT6B::ADK* #3	AGAAAGACCAGAAAGTGAAGT
sh*KAT6B::ADK* #4	GAAAGACCAGAAAGTGAAGTC
sh*KAT6B::ADK* #5	AAAGACCAGAAAGTGAAGTCA
sh*KAT6B::ADK* #6	AAGACCAGAAAGTGAAGTCAC
sh*KAT6B::ADK* #7	TGAGAAAGACCAGTGGATGAT
sh*KAT6B::ADK* #8	GAGAAAGACCAGTGGATGATT
sh*KAT6B::ADK* #9	AGAAAGACCAGTGGATGATTC
sh*KAT6B::ADK* #10	GAAAGACCAGTGGATGATTCA
sh*KAT6B::ADK* #11	AAAGACCAGTGGATGATTCAA
sh*KAT6B::ADK* #12	AAGACCAGTGGATGATTCAAC

Supplementary Table S3. List of antibodies used in this study

Antibodies	SOURCE	IDENTIFIER
Mouse anti-human GFP	Abclonal	Cat# AE012
Rabbit anti-human ATF4	Affinity	Cat# DF6008
Rabbit anti-human ATF3	Selleck	Cat #F0316
Rabbit anti-human eIF2a	Selleck	Cat #F1121
Rabbit anti-human phospho-eIF2a (Ser51)	Affinity	Cat #AF3087
Rabbit anti-human GCN2	Affinity	Cat# DF7801
Rabbit anti-human phospho-GCN2 (Thr899)	Abclonal	Cat# AP1356
Rabbit anti-human ADK	Abclonal	Cat# A15023
Mouse anti-human Vinculin	Abclonal	Cat# A2752
